# Novel Regioisomeric Analogues of Naphthyl-*N*-Acylhydrazone Derivatives and Their Anti-Inflammatory Effects

**DOI:** 10.3390/ijms232113562

**Published:** 2022-11-05

**Authors:** Dayana da Costa Salomé, Rosana Helena Coimbra Nogueira de Freitas, Carlos Alberto Manssour Fraga, Patricia Dias Fernandes

**Affiliations:** 1 Laboratório de Farmacologia da Dor e da Inflamação, Programa de Pesquisa em Descoberta de Fármacos, Instituto de Ciências Biomédicas, Universidade Federal do Rio de Janeiro, Rio de Janeiro 21941-901, Brazil; 2 Laboratório de Avaliação e Síntese de Substâncias Bioativas (LASSBio), Programa de Pesquisa em Descoberta de Fármacos, Instituto de Ciências Biomédicas, Universidade Federal do Rio de Janeiro, Rio de Janeiro 21941-901, Brazil

**Keywords:** anti-inflammatory substance, *N*-acylhydrazone, naphthyl-*N*-acylhydrazone, LASSBio-1524 analogues

## Abstract

Background: When homeostasis is disturbed it can result in a pathological event named inflammation. The main drugs used in the treatment consist of non-steroidal and steroidal anti-inflammatory drugs. However, the side effects remain an obstacle during the treatments. In this study, we aimed to evaluate three new regioisomers analogues of naphthyl-*N*-acylhydrazone derivatives. Methods: Acute models of inflammation in vivo (formalin-induced licking and carrageenan-induced inflammation) as well as in vitro were used to evaluate the effects of LASSBio-2039, LASSBio-2040, and LASSBio-2041. Results: All three substances (at 1, 10 or 30 µmol/kg) presented significant effects in the in vivo model reducing leukocyte migration, nitric oxide (NO) and interleukin-1β production. It was observed that only LASSBio-2039 significantly reduced cell migration in vitro. None of the LASSBios affected inducible nitric oxide synthase activity nor presented nitric oxide (NO) scavenger effect. No toxic effect was observed, either in vivo or in vitro. The new regioisomers analogues of naphthyl-*N*-acylhydrazone derivatives presented significant anti-inflammatory activity, suggesting LASSBio-2039 has a direct effect in leukocytes migratory capacity. Conclusions: Taken together, the data indicate that these substances present promising effects for the development of a prototype for new drugs.

## 1. Introduction

The definition of inflammation has been continuously adapted. Recently, it was characterized as a tissue response to an emergent stimulus. This reaction can be macro and/or microscopically identified and there is involvement of a variety of cells. Altogether, these events can lead to necrosis, edema, fibrosis, malignancy and/or infection. An excessive inflammatory response not controlled can evolve into several diseases such as arthritis, osteoporosis [1], asthma [2], Alzheimer’s disease [3], cardiovascular disease [4], cancer [5] and obesity [6]. Thus, the search and development of new anti-inflammatory substances that could reduce or eliminate the inflammatory process continues to be an objective for several groups. Although there is a wide variety of anti-inflammatory drugs, there are concomitantly a wide variety of side effects that can limit their use. Thus, the continuous search for new chemical entities with anti-inflammatory potential and lower incidence of side effects remains a goal for researchers in this area.

Derivatives LASSBio-1524 (1) and LASSBio-1760 (2) have already been described as powerful anti-inflammatory prototypes with action in several acute and chronic models of inflammation [7,8,9]. These two compounds present the *N*-acylhydrazone (NAH) subunit, widely described as a privileged subunit [10], useful for discovering new drug candidates due to its peptidomimetic nature and superior stability to chemical and metabolic hydrolysis [11]. 

**Figure 1:**
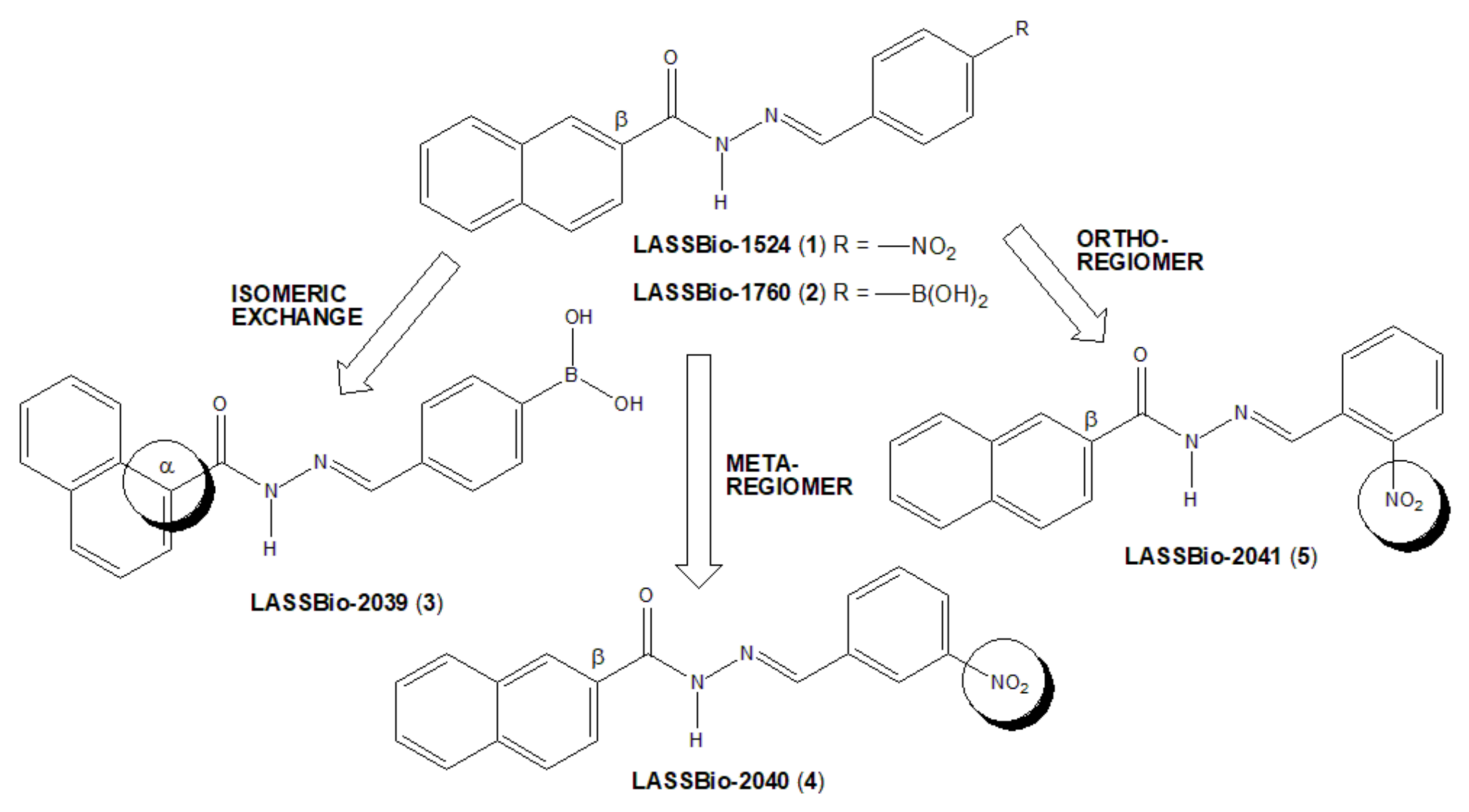
Design concept of novel naphthyl-*N*-acylhydrazone derivatives (**3–5**).

The bioisosteric relationship of 4-nitrophenyl fragment by 4-phenylboronic acid subunit was previously characterized by comparing the anti-inflammatory profiles of compounds 1 and 2 [8]. An isomeric exchange was performed in the naphthyl subunit of LASSBio-1760 (2), with NAH linked to position α of the naphthyl subunit to generate LASSBio-2039 (3) (Figure 1). Additionally, two LASSBio-1524 (1) regioisomers were proposed, with the exchange of the nitro group of LASSBio-1524 from para-position to meta position (LASSBio-2040, 3) or to the ortho position (LASSBio-2041, 5).

The changes introduced in new regioisomeric analogues (**4**) and (**5**) were purposed to avoid the potential toxicity produced by nitroaromatic derivatives [12]. The main idea was exchanging the position of the nitro group from a more accessible para-position to a more hindered meta- and ortho-position, respectively, in order to prevent access to the CYP reductase enzyme [13]. Moreover, it is well-known from the literature that the β-substituted naphthyl group is more susceptible to oxidative metabolism to form toxic metabolites than the corresponding α-substituted naphthyl, as learned from the discovery of β-blocker propranolol from its precursor pronetalol [14]. So, we proposed the exchange of the β-naphthyl group present in LASSBio-1760 (**2**) to the α-naphthyl group in LASSBio-2039 (**3**), in order to reduce the potential toxicity of this new drug candidate.

Thus, in this study, we described the synthesis and anti-inflammatory actions of a new small series of naphthyl-*N*-acylhydrazones (**3**–**5**), planned as regioisomeric analogues of LASSBio-1524 (**1**) and LASSBio-1760 (**2**).

## 2. Results

### 2.1. Chemistry

**Scheme 1:**
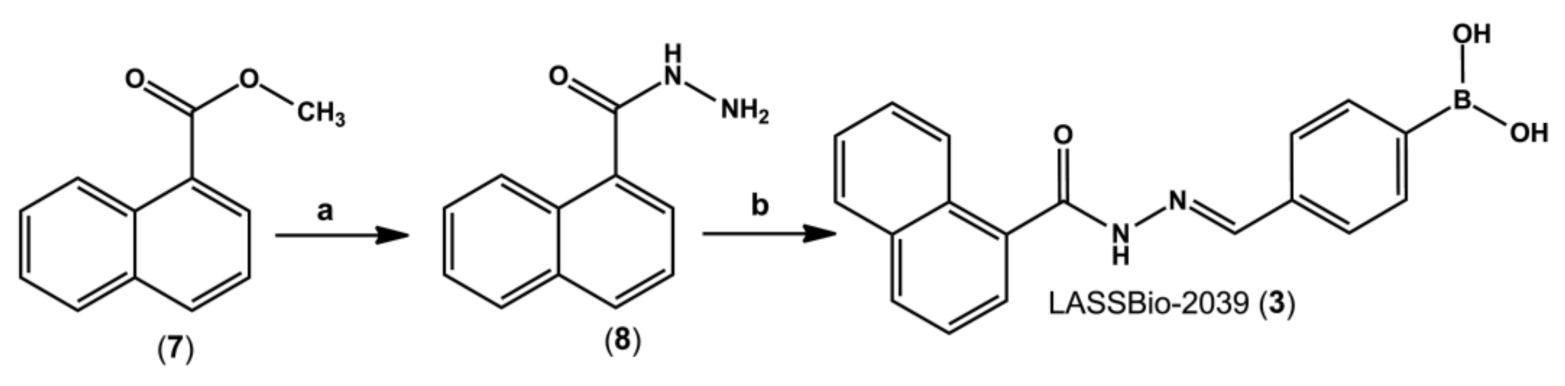
Reagents and conditions: (**a**) NH_2_NH_2_.H_2_O 100%, EtOH, reflux, 6 h, 83%; (**b**) 4-formylphenylboronic acid, EtOH, cat. HCl 20%, rt, 3 h, 92%.

LASSBio-2039 (**3**) were synthesized from 1-naphtoic acid, which underwent Fischer esterification reaction in methanol, sulfuric acid at reflux to generated methyl 1-naphthoate (**7**) [15,16]. Next, hydrazinolysis reaction of ester (**7**), performed by reflux of an ethanolic solution containing anhydrous hydrazine hydrate, generated 1-naphthohydrazide (**8**) in 70% yield [17,18]. The final step consisted of acid catalyzed condensation of 1-naphthohydrazide (**8**) with 4-formylphenylboronic acid to furnish *N*-acylhydrazone LASSBio-2039 (**3**) in 92% yield [8] (Scheme 1).

**Scheme 2:**
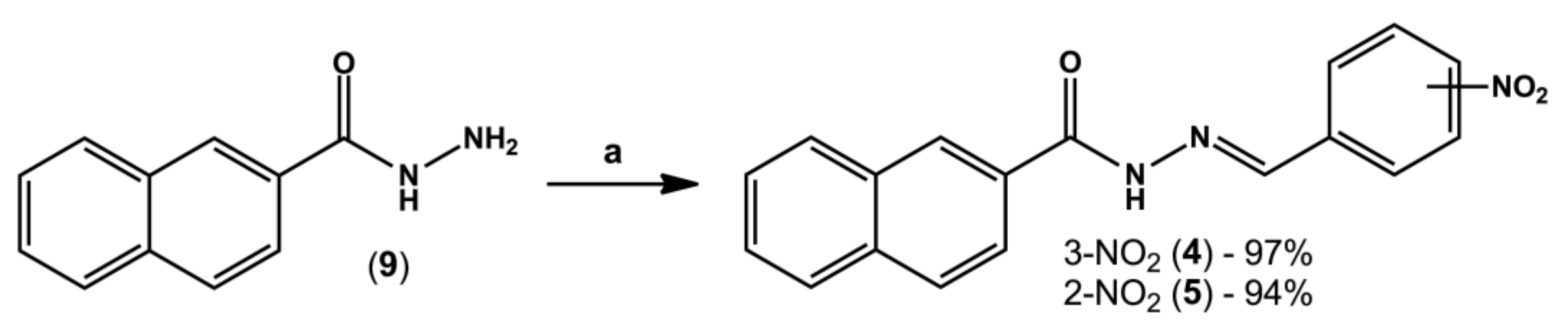
Reagents and conditions: (a) 3-nitrobenzaldehyde or 2-nitrobenzaldehyde, EtOH, cat. HCl 20%, rt, 3 h, 94–97%.

2-Naphthohydrazide (**9**), the key intermediate for the synthesis of compounds **4** and **5**, was prepared as previously described by Cordeiro et al. [8]. So, NAH derivatives **4** and **5** were obtained by adding, respectively, 3- or 2-nitrobenzaldehyde to 2-naphthohydrazide (**9**) under acid catalysis and at room temperature [17]. Both *N*-acylhydrazones (**4**–**5**) were obtained in very high yields as described in Scheme 2.

All NAH derivatives were obtained as a single diastereoisomer, the most stable, (*E*)-diastereoisomer. This statement can be confirmed and corroborated through the analysis of ^1^H-NMR spectra of molecules, which shows signs referring to only one imine hydrogen. Furthermore, this spectroscopic characteristic was already described in the literature, and it is a strong indication of the formation of (*E*)-diastereoisomer [19,20,21].

The obtained *N*-acylhydrazone derivatives (**3**–**5**) were fully spectroscopically characterized and their degree of purity was determined by reversed-phase HPLC analysis to be greater than 95%, which was considered adequate for the next step of investigating their antinociceptive and anti-inflammatory actions.

### 2.2. LASSBios Did Not Induce Any In Vitro or In Vivo Toxic Effect

**Figure 2:**
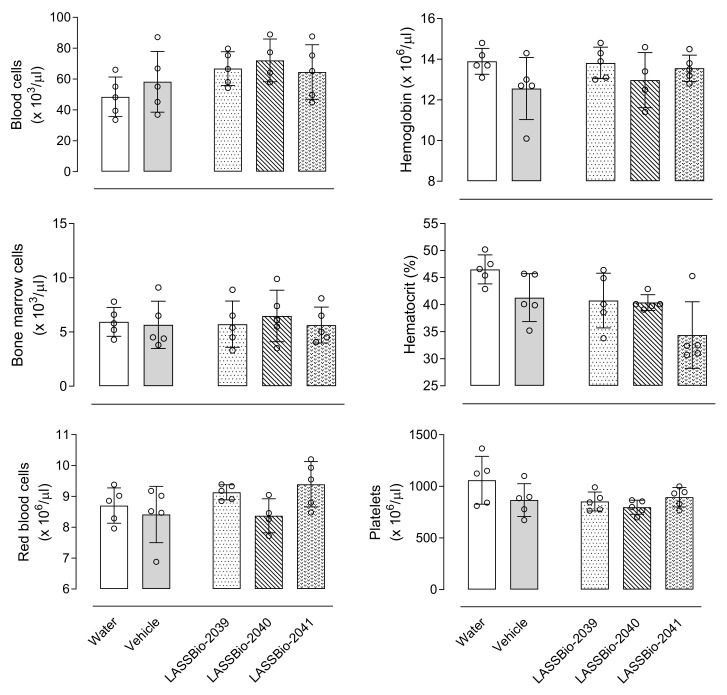
LASSBio-2039, LASSBio-2040 and LASSBio-2041 did not affect blood, bone marrow or red blood cells or platelet count nor hemoglobin and hematocrit levels. Mice were orally treated with each of the substances (30 µmol/kg), water or vehicle. After 24 h bone marrow and blood were collected to measurements. Results are expressed as mean ± standard deviation (*n* = 5–7).

Pretreatment of mice with a single oral dose of any of LASSBios (at 30 µmol/kg) did not affect either bone marrow cell count or hemogram values (blood leukocyte cell count, hematocrit, red blood cell count, hemoglobin, and hematocrit values) (Figure 2). 

**Figure 3:**
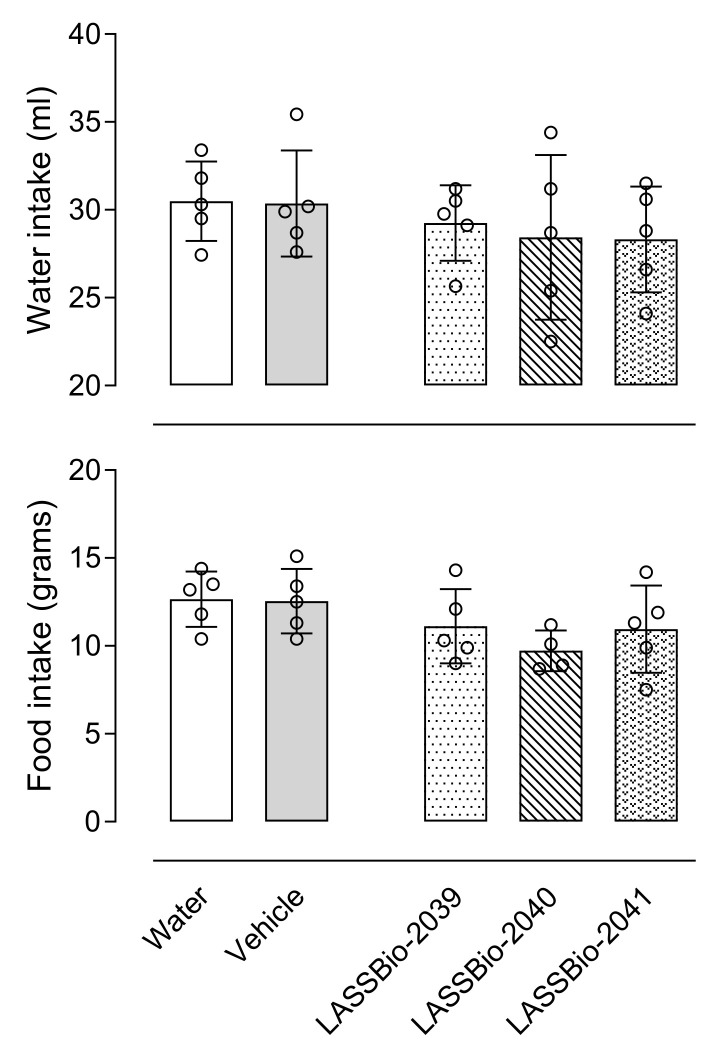
LASSBio-2039, LASSBio-2040 and LASSBio-2041 did not affect food and water intake. Mice were orally treated with each of the substances (30 µmol/kg), water or vehicle. After 24 h the amount of water (in mL) or food (in grams) ingested were evaluated. Results are expressed as mean ± standard deviation (*n* = 5–7).

We did not observe alterations to respiration and no ulcers were observed in the stomach after 5 days. Additionally, there were no alterations in normal activity, such as food and water intake (Figure 3), grooming, and loss of righting reflex. The incubation of J774.A1 macrophage cell line with concentrations of 1, 10 or 30 µM of each LASSBio did not affect cell viability, even after 24 h incubation.

### 2.3. LASSBio-2039, LASSBio-2040 and LASSBio-2041 Did Present Antinociceptive Effect in an Inflammatory Pain Model

**Figure 4:**
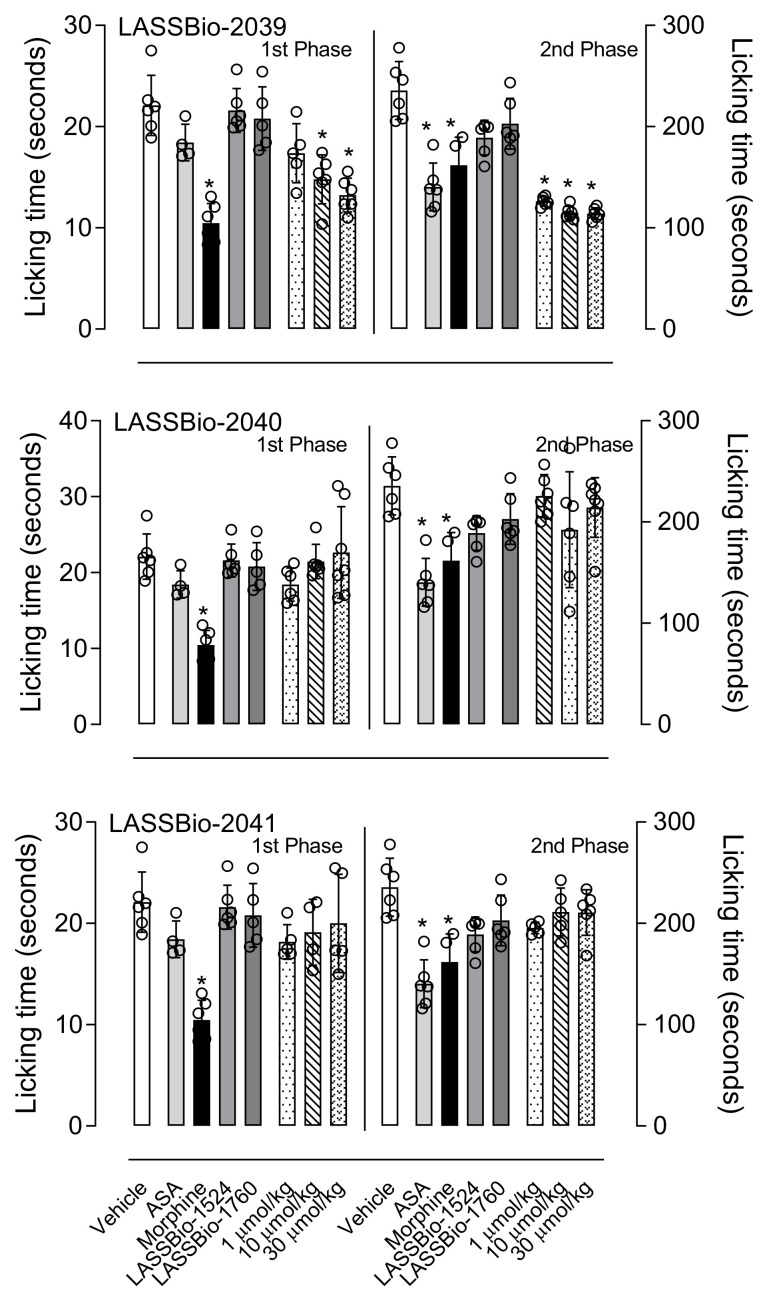
LASSBio-2039, LASSBio-2040 and LASSBio-2041 effects in an inflammatory pain model. Mice were orally treated with each of the substances with three different doses, acetylsalicylic acid (1100 µmol/kg), morphine (15 µmol/kg), LASSBio-1524 or LASSBio-1760 (30 µmol/kg) or vehicle. One hour later the nociceptive response was induced by intraplantar injection of formalin (2.5%) in right hind paw. Results are expressed as mean ± standard deviation (*n* = 5–7). Statistical analyses were calculated in GraphPad Prism 8.0 (San Diego, CA, USA) using analyses of variance (ANOVA) followed by Tukey post-test with *p* < 0.01 (*) when comparing treated groups with vehicle-treated group. Data for the original compounds (LASSBio-1524 and LASSBio-1760) are original, independent duplicates of past results and have not been published previously.

Figure 4 shows the effects of pretreatment of mice with LASSBio-2039, LASSBio-2040 or LASSBio-2041 one hour before formalin injection into the hind paw. Mice receiving the vehicle remained linking the formalin-injected paw during 22 ± 3 s and 2356 ± 29 s, for the first and second phases, respectively. The pretreatment of animals with acetylsalicylic acid (ASA, 1100 µmol/kg) or morphine (15 µmol/kg) resulted in 14% and 53% reduction in the first phase, respectively, and an inhibition in 40% and 15% in the second phase, respectively.

When LASSBio-2039 (1, 10 or 30 µmol/kg) was used, a reduction of 38%, 45% and 48%, respectively, was observed in the licking response of the first phase and 46%, 51% and 51% inhibition in the second phase of the model. When compared with the original substance, LASSBio-1760, similar effects can be observed. It is important to note that neither LASSBio-2040 nor LASSBio-2041 affected the time of reaction in any dose tested. 

### 2.4. LASSBios Reduced Inflammatory Parameters in an Acute Model of Inflammation

**Figure 5:**
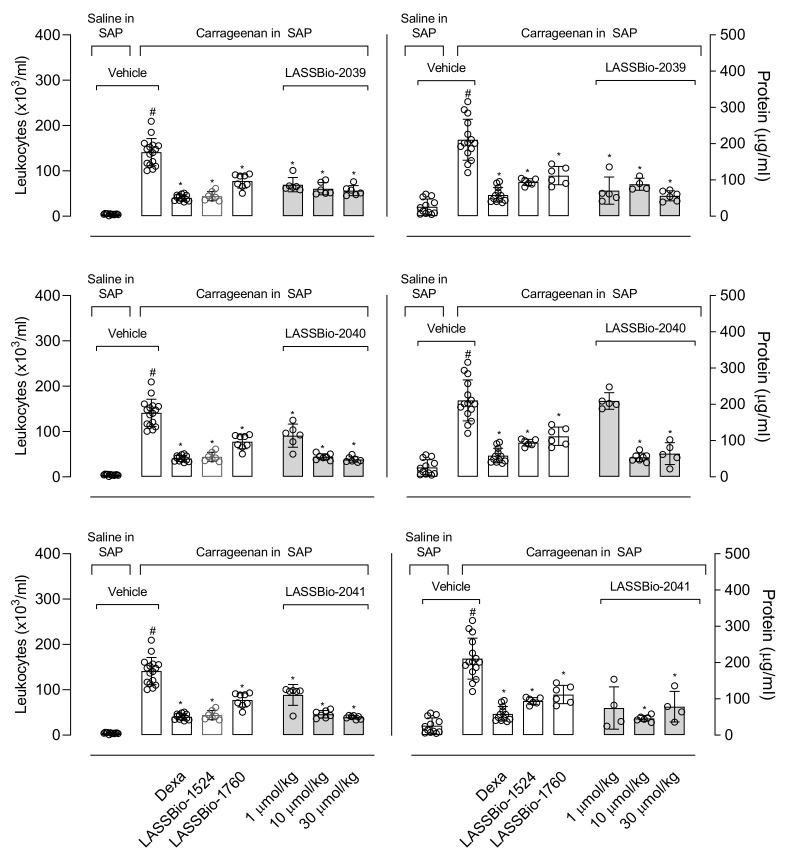
LASSBios reduce cell migration induced by carrageenan into the subcutaneous air pouch (SAP) and protein extravasated. Animals were pre-treated with vehicle, dexamethasone (6.5 µmol/kg) or LASSBios (doses of 1, 10 or 30 µmol/kg) 1 h before injection of saline (NaCl 0.9%) or carrageenan into the SAP. Results are expressed as media ± SD (*n* = 5–12). Statistical significance was calculated in GraphPad Prism 8.0 (San Diego, CA, USA) using analyses of variance (ANOVA) followed by Tukey post-test with *p* < 0.01 (#) when comparing vehicle-treated group that received carrageenan injection into the SAP with vehicle-treated group that receives saline into the SAP or *p* < 0.01 (*) when comparing dexamethasone- or LASSBios-treated group that received carrageenan injection into the SAP with vehicle-treated group that receives carrageenan into the SAP. Data for the original compounds (LASSBio-1524 and LASSBio-1760) are original, independent duplicates of past results and have not been published previously.

The injection of carrageenan (0.5%) into the subcutaneous air pouch induced a 35-fold increase in leukocyte migration toward the pouch (167.3 ± 25.5 × 10^6^ cells/mL versus 4.6 ± 1.9 × 10^6^ cell/mL in animals that receives only saline in SAP). Dexamethasone (a steroidal anti-inflammatory drug, 6.5 µmol/kg) significantly reduced in 72% the number of leukocytes in the SAP (34 ± 2 × 10^6^ cells/mL). When mice were pre-treated with LASSBio-2039 (1, 10 or 30 µmol/kg), a dose-dependent reduction was observed (58%, 63% and 66%, respectively) in cell migration. Similarly, both LASSBio-2040 and LASSBio-2041 presented significant effects. LASSBio-2040 reduced by 48%, 69% and 73%; LASSBio-2041 inhibited in 29%, 62% and 68% for the doses of 1, 10, 30 µmol/kg, respectively. It is interesting to note that the dose of 1 µmol/kg of LASSBios presented an inhibitory effect similar to that observed with dexamethasone. When compared with the original substances (LASSBio-1524 and LASSBio-1760, at 30 µmol/kg), it can be noted that the new compounds were most effective in reducing cell migration than the original molecules, since the lower dose used showed an effect comparable to the dose used from the LASSBio-1524 and LASSBio-1760 (Figure 5, left graphs).

We next decided to evaluate the capacity of each of the LASSBios in reducing protein extravasation induced by carrageenan injected in the SAP. This phlogistic agent induced a 7-fold increase in the amount of protein extravasated to the exudate (212.6 ± 50.7 µg/mL protein in carrageenan-injected group versus 33.7± 19.3 µg/mL in vehicle-treated group receiving saline in the SAP). Pre-treatment of mice with dexamethasone caused a 71% reduction in the protein extravasated. It is interesting to observe that all three LASSBios significantly reduced protein extravasation with exception to a lower dose of LASSBio-2040. When comparing LASSBios, the most potent was LASSBio-2039. All three substances were also more potent than the original compound (Figure 5, right graphs). 

### 2.5. LASSBios Inhibited Cytokines Production

**Figure 6:**
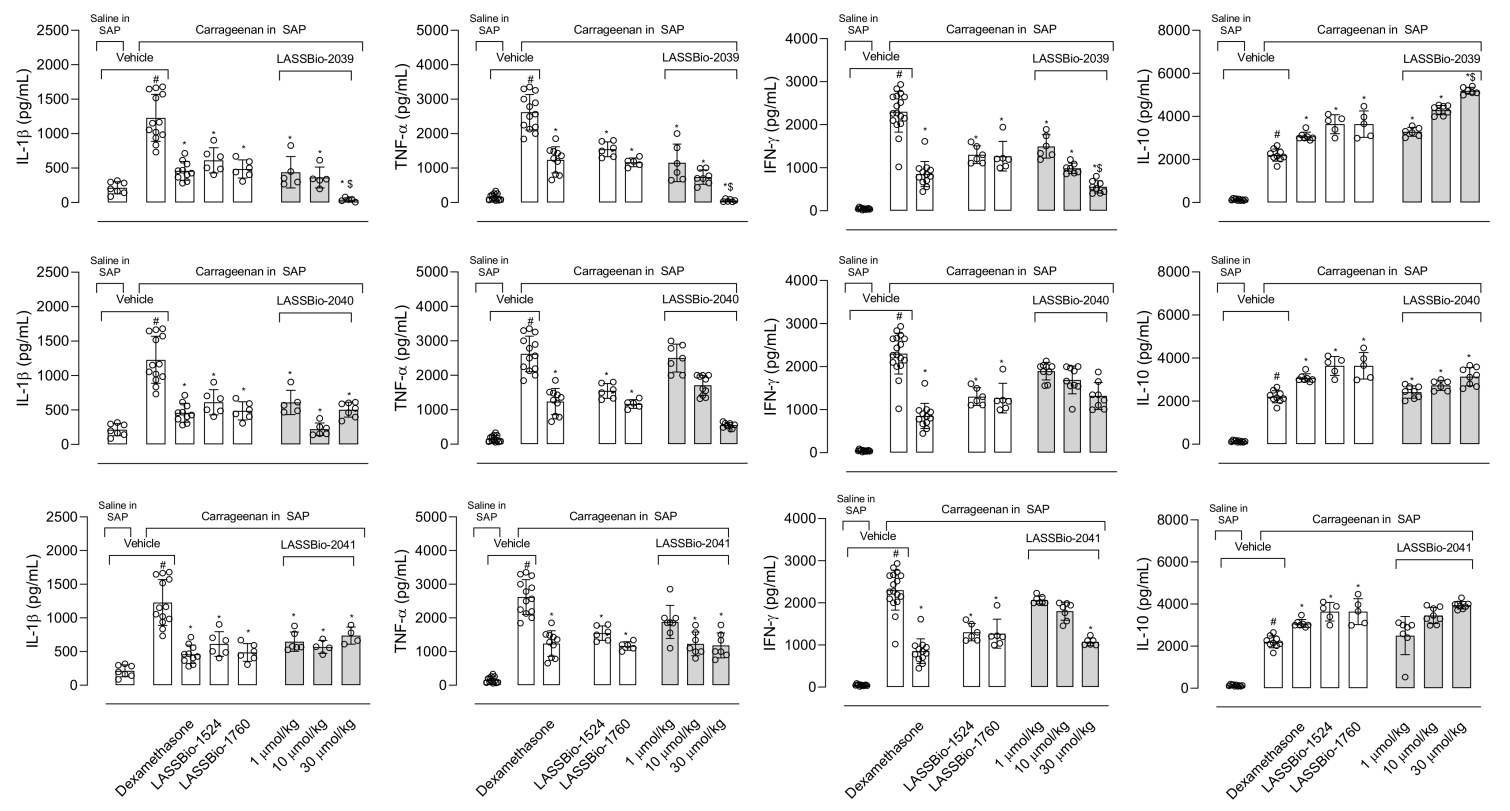
LASSBios reduce interleukin-1β (IL-1β), tumor necrosis factor-α (TNF-α), interferon-γ (IFN-γ) and increase interleukin-10 (IL-10) produced in the inflammatory exudate. Animals were pre-treated with vehicle, LASSBio-1524 or LASSBio-1760 (at 30 µmol/kg), dexamethasone (6.5 µmol/kg) or LASSBios (doses of 1, 10 or 30 µmol/kg) 1 h before injection of saline (NaCl 0.9%) or carrageenan into the SAP. Results are expressed as media ± SD (*n* = 5–12). Where no error bar is shown it is because it is smaller than the symbol. Statistical significance was calculated in GraphPad Prism 8.0 (San Diego, CA, USA) using analyses of variance (ANOVA) followed by Tukey post-test with *p* < 0.01 (#) when comparing vehicle-treated group that received carrageenan injection into the SAP with vehicle-treated group that receives saline into the SAP. *p* < 0.01 (*) when comparing dexamethasone- or LASSBios-treated group that received carrageenan injection into the SAP with vehicle-treated group that receives carrageenan into the SAP. *p* < 0.01 ($) when comparing LASSBio-2039-treated group that received carrageenan injection into the SAP with LASSBio-1760-treated group that receives carrageenan into the SAP. Data for the original compounds (LASSBio-1524 and LASSBio-1760) are original, independent duplicates of past results and have not been published previously.

Figure 6 shows that LASSBio-2039 significantly and dose-dependently reduced interleukin-1β (IL-1β), tumor necrosis factor-α (TNF-α) and interferon-γ (INF-γ) production. The dose of 30 µmol/kg almost completely abolished cytokines production with values close to those observed in the control group. An increase in interleukin-10 (IL-10) production in those group of mice pretreated with LASSBio-2039 was also observed. It is interesting to note that pretreating mice with the higher dose resulted in a significant effect, even when compared with dexamethasone-treated mice. In those groups of mice pretreated with LASSBio-2040 or LASSBio-2041, a reduction in all three cytokines production was observed. Although in some groups these effects were significant, the inhibition caused by both compounds was not as intense when compared with LASSBio-2039.

**Figure 7:**
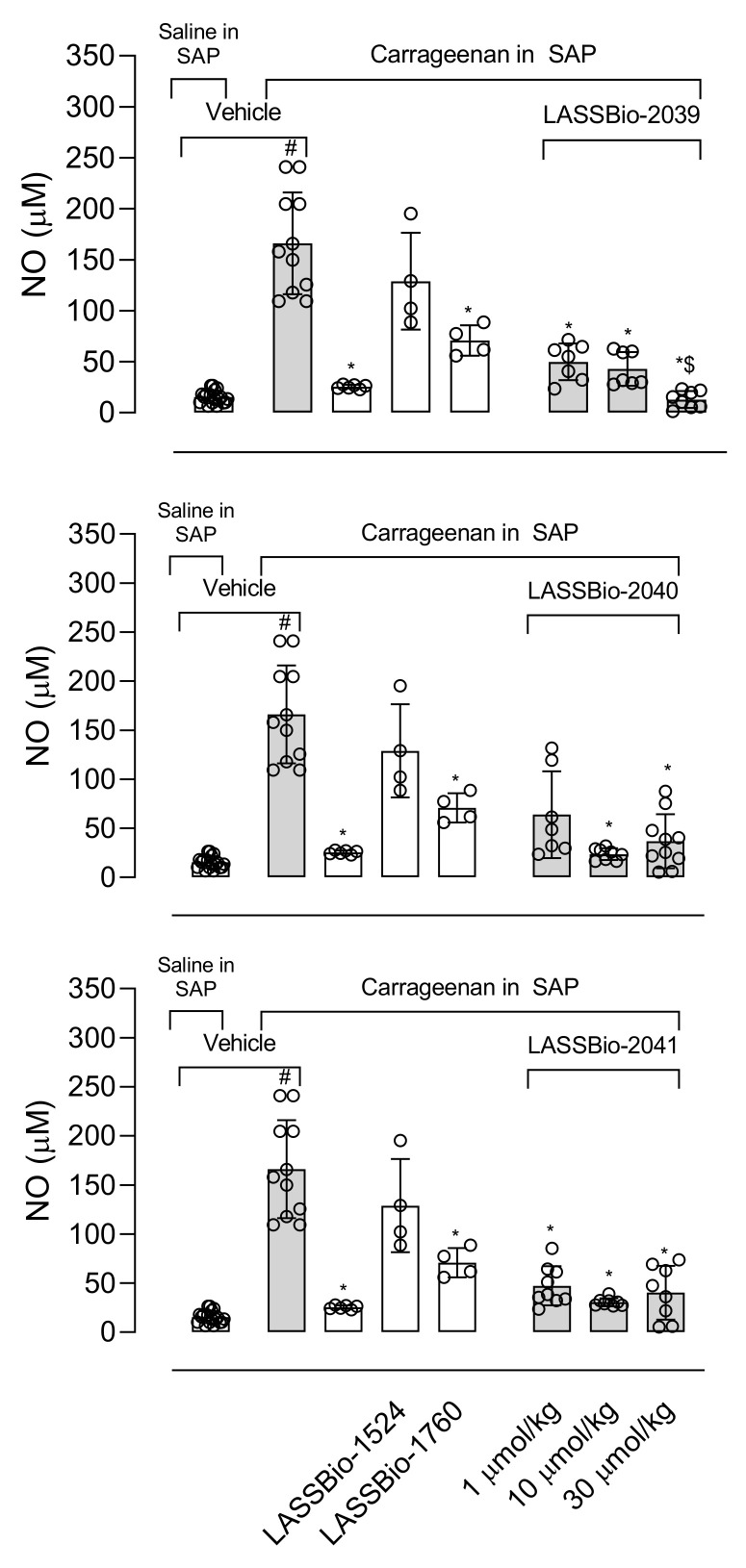
LASSBios reduce nitric oxide (NO) produced in the inflammatory exudate. Animals were pre-treated with vehicle, LASSBio-1524 or LASSBio-1760 (at 30 µmol/kg), dexamethasone (6.5 µmol/kg) or LASSBios (doses of 1, 10 or 30 µmol/kg) 1 h before injection of saline (NaCl 0.9%) or carrageenan into the SAP. Results are expressed as media ± SD (*n* = 5–12). Where no error bar is shown it is because it is smaller than the symbol. Statistical significance was calculated in GraphPad Prism 8.0 (San Diego, CA, USA) using analyses of variance (ANOVA) followed by Tukey post-test with *p* < 0.01 (#) when comparing vehicle-treated group that received carrageenan injection into the SAP with vehicle-treated group that receives saline into the SAP. *p* < 0.01 (*) when comparing dexamethasone- or LASSBios-treated group that received carrageenan injection into the SAP with vehicle-treated group that receives carrageenan into the SAP. *p* < 0.01 ($) when comparing LASSBio-2039-treated group that received carrageenan injection into the SAP with LASSBio-1760-treated group that receives carrageenan into the SAP. Data for the original compounds (LASSBio-1524 and LASSBio-1760) are original, independent duplicates of past results and have not been published previously.

We also measured the amount of nitric oxide (NO) produced in the inflammatory exudate. NO is an instable mediator that is rapidly conversed and decayed to nitrate when in biological fluids. In normal conditions, the level of this mediator is very low, as we can observe in the groups of animals that received saline injection in the SAP (15.4 ± 6.2 µM of NO). However, after an inflammatory insult such as carrageenan, a 12-fold increase was observed. The total amount of NO measured in exudates obtained from the carrageenan group was 189.2 ± 45.4 µM. The pre-treatment of mice with dexamethasone resulted in an 86% reduction in NO produced. Our data show that LASSBio-2039 reduced by at least 75%, and the higher dose (30 µmol/kg) completely blocked the production of NO. When evaluating the results obtained with LASSBio-2040 and LASSBio-2041, it was observed that even with 1 µmol/kg, the reduction in NO production was 54% and 71%, respectively. The other two doses inhibited at least 70% of the mediator production. When compared with the original compounds, LASSBio-1524 and LASSbio-1760, data demonstrated the LASSBio-2039 was more potent than LASSbio-1760 in reducing NO production (at 30 µmol/kg) (Figure 7).

### 2.6. LASSBios Also Reduced Inflammatory Parameters In Vitro

**Figure 8:**
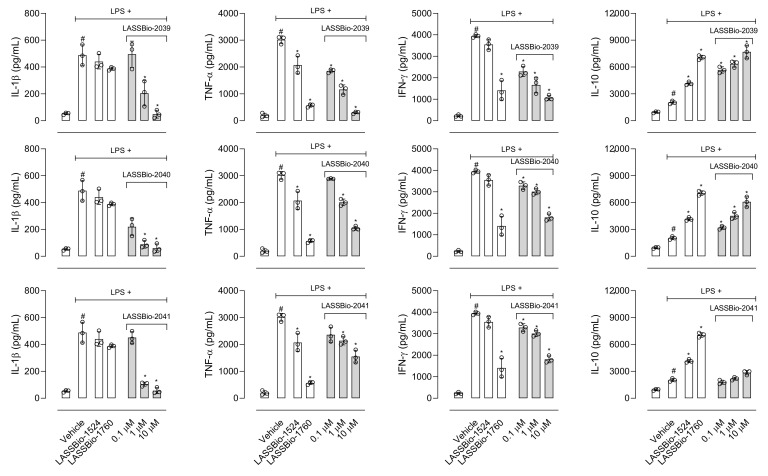
Effect of LASSBios in interleukin-1β (IL-1β), tumor necrosis factor-α (TNF-α), interferon-γ (IFN-γ) and interleukin-10 (IL-10) produced by J774.A1 cells line. Cells were incubated with vehicle, LASSBio-1524 or LASSBio-1760 (at 30 µM), or LASSBios (0.1, 1 or 10 µM) and after 1 h were activated with LPS (1 µg/mL). Results are expressed as media ± SD (*n* = 3). Where no error bar is shown it is because it is smaller than the symbol. Statistical significance was calculated in GraphPad Prism 8.0 (San Diego, CA, USA) using analyses of variance (ANOVA) followed by Tukey post-test with *p* < 0.01 (#) when comparing LPS-activated cells treated with vehicle and non-activated cells treated with vehicle. *p* < 0.01 (*) when comparing LPS-activated cells treated with LASSBios and LPS-activated cells treated with vehicle. Data for the original compounds (LASSBio-1524 and LASSBio-1760) are original, independent duplicates of past results and have not been published previously.

Data obtained using in vivo models indicated that all three LASSBios significantly reduced leukocyte migration and the production of cytokines and NO. These effects could be a direct result of the reduction in cell viability and/or reduction in the number of cells arriving in the inflammatory site, thus resulting in a reduced number of cells producing the mediators. To rule out these possibilities, we used a macrophage cell line (J774.A1) activated with lipopolysaccharide (LPS) and measured the production of NO, IL-1β, TNF-α, IFN-γ and IL-10. As can be observed in Figure 8, non-LPS-activated cells produced low levels of cytokines. However, when activated with LPS, there was an increase of at least 10-fold.

Similar to data obtained in the SAP model, LASSBio-2039 (at 10 µM) almost completely inhibited IL-1β and TNF-α production. It was also observed that LASSBio-2040 and LASSBio-2041 significantly inhibited cytokines production. However, these effects were not as intense as those observed in the SAP model, suggesting that those effects could be a group of actions (reduction in cell migration and a direct effect in cytokines production by migrated cells).

**Figure 9:**
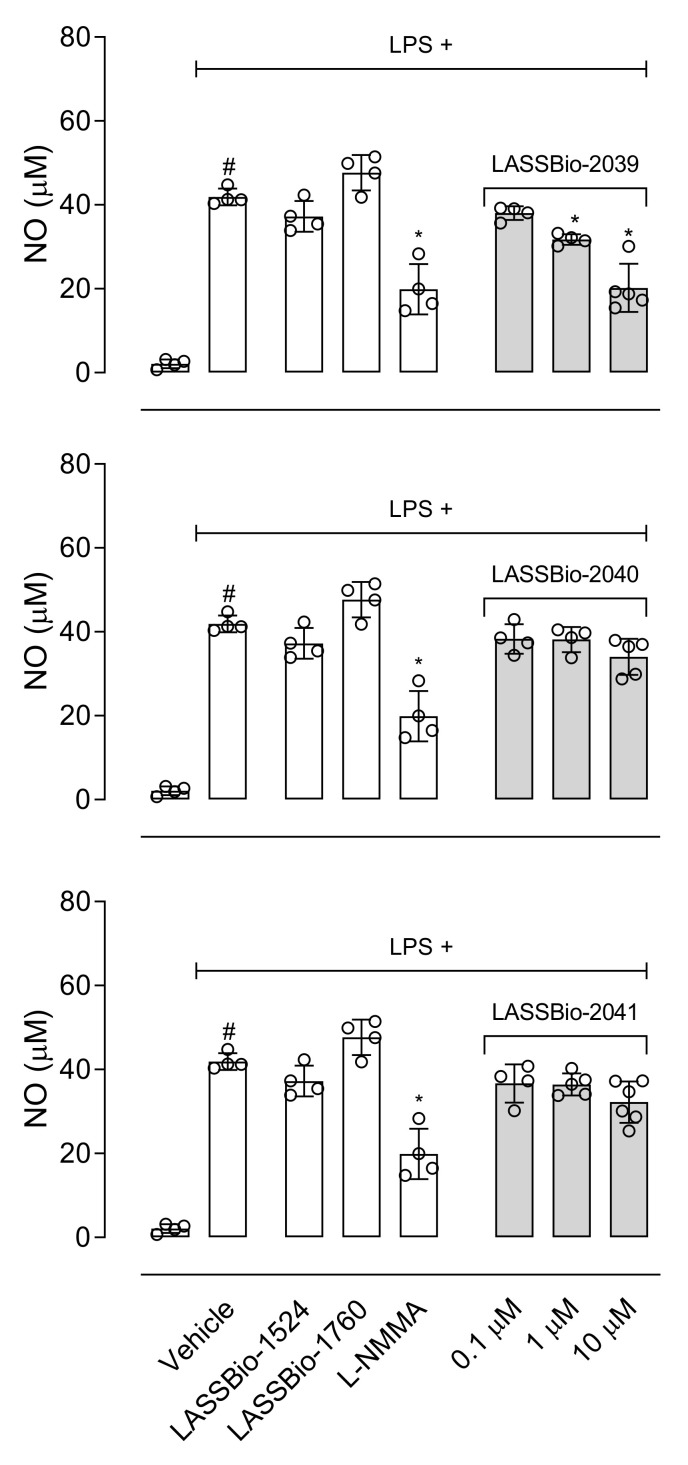
Effect of LASSBios in nitric oxide (NO) produced by J774.A1 cells line. Cells were incubated with vehicle, L-nitro mono methyl arginine (L-NMMA) or LASSBios (0.1, 1 or 10 µM) and after 1 h were activated with LPS (1 µg/mL). NO was measured 24 h later. Results are expressed as media ± SD (*n* = 4–6). Where no error bar is shown is because it is smaller than the symbol. Statistical significance was calculated in GraphPad Prism 8.0 (San Diego, CA, USA) using analyses of variance (ANOVA) followed by Tukey post-test with *p* < 0.01 (#) when comparing LPS-activated cells treated with vehicle and non-activated cells treated with vehicle. *p* < 0.01 (*) when comparing LPS-activated cells treated with LASSBios and LPS-activated cells treated with vehicle. Data for the original compounds (LASSBio-1524 and LASSBio-1760) are original, independent duplicates of past results and have not been published previously.

Then, we evaluated the ability of each compound to inhibit NO production in vitro. The data obtained (Figure 9) show that although LASSBio-2039 significantly reduced NO production when LPS-activated cells were incubated with 1 and 10 µM, neither LASSBio-2040 nor LASSBio-2041 affected NO production with any of the concentrations used. 

**Figure 10:**
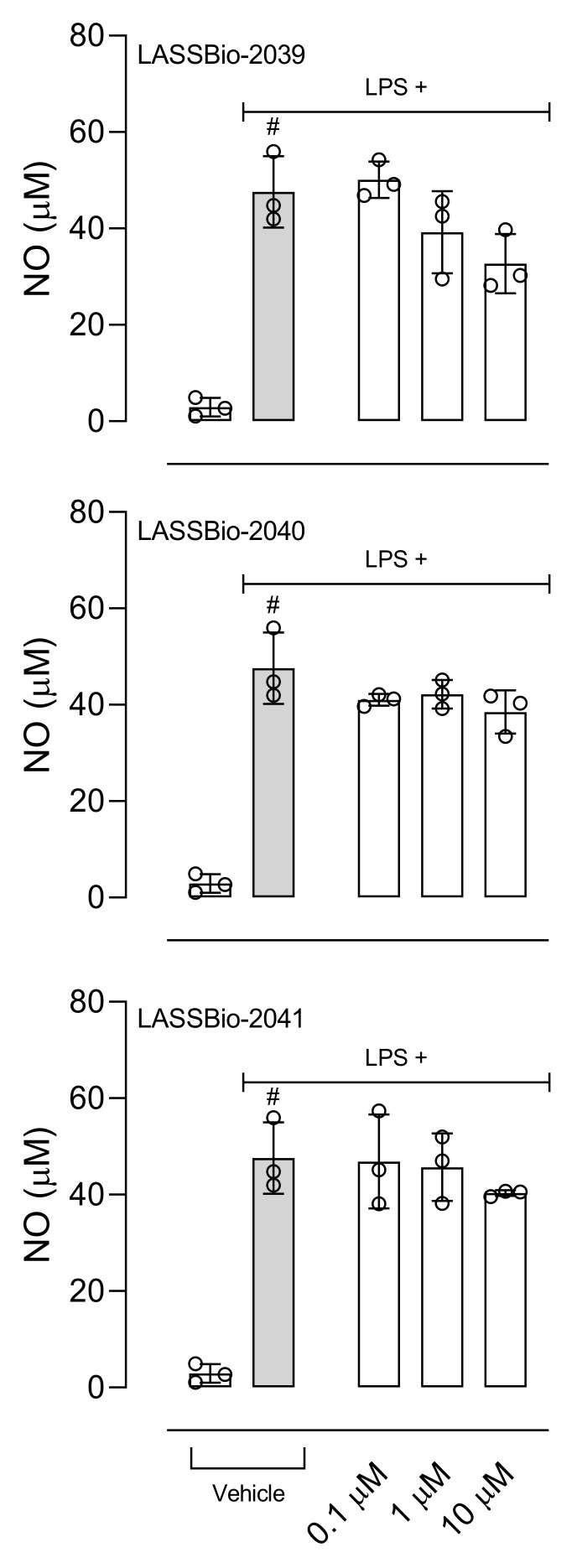
Effect of LASSBios in inducible nitric oxide synthase enzyme activity. J774.A1 cells were incubated with vehicle or lipopolysaccharide (LPS, 1 µg/mL) and after 8 h received LASSBios at different concentrations (0.1, 1 or 10 µM). Twenty-four hours after LPS activation NO accumulated in supernatant was measured. Results are expressed as media ± SD (*n* = 3). Where no error bar is shown it is because it is smaller than the symbol. Statistical significance was calculated in GraphPad Prism 8.0 (San Diego, CA, USA) using analyses of variance (ANOVA) followed by Tukey post-test with *p* < 0.01 (#) when comparing LPS-activated cells treated with vehicle and non-activated cells treated with vehicle.

The data obtained so far are suggestive that LASSBio-2039 can inhibit NO production; however, we cannot conclude if this effect is due to inhibition in inducible nitric oxide synthase (iNOS) expression, its activity, or a direct NO-scavenger effect of each substance. Trying to elucidate these possibilities, we first incubated LPS-activated J774.A1 cells with LASSBios and after 8 h of activation, a period where protein synthesis of iNOS was finished and the enzyme begins it activity, LASSBios were added to culture medium. After 24 h of activation, the supernatants were collected, and NO was measured. Results shown in Figure 10 demonstrated that none of LASSBios affected the NO production when added 8 h post-LPS activation, suggesting that their effects do not occur in enzyme activity. 

To rule out the possibility that LASSBios could act as a NO scavenger due to a direct interaction with the gas immediately after its production by cells, the NO donor SNAP was incubated with LASSBios. It was observed that none of substances showed ability in scavenger NO, thus reducing the levels of nitrite measured in the medium.

### 2.7. LASSBio-2039 Did Reduce Cell Migration In Vitro

**Figure 11:**
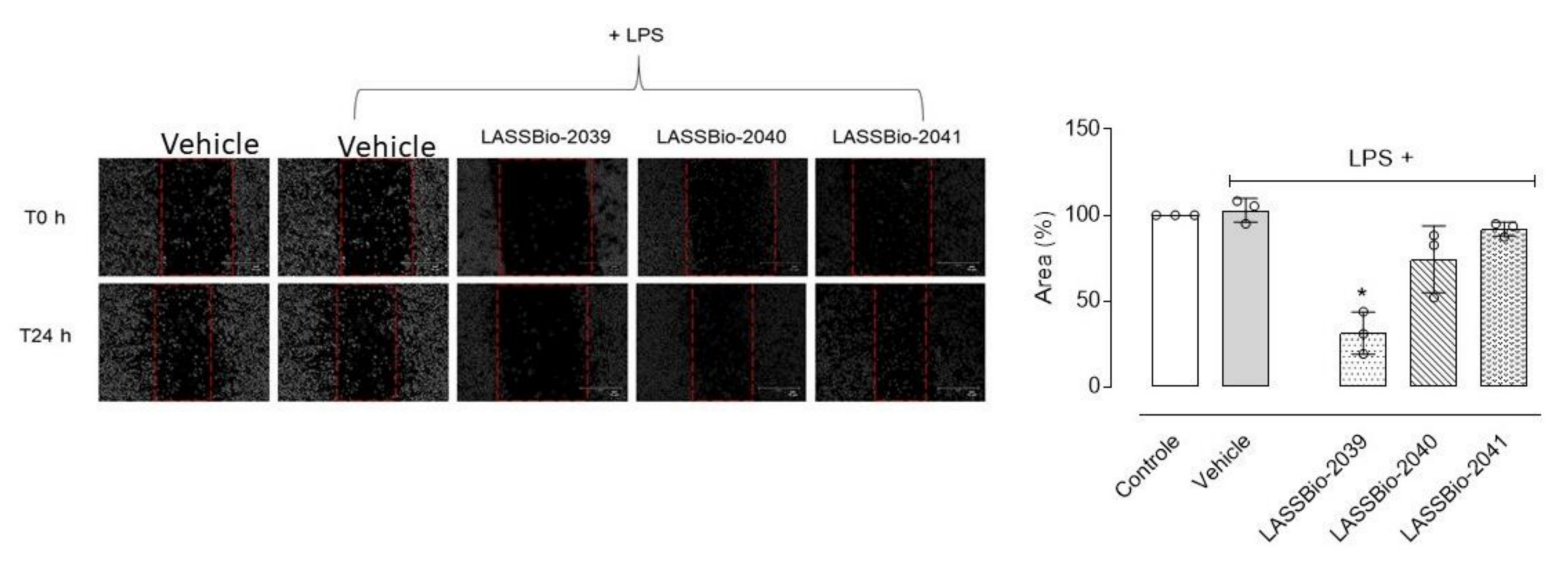
Effect of LASSBios in cell migration in vitro. J774.A1 cells were plated, and a wound area done. Cells were incubated with vehicle or LASSBios (10 µM) 15 min before lipopolysaccharide (LPS, 1 µg/mL) and after 24 h the area in each well was measured with the aid of ImageJ software (version 1.8.0.172, National Institute of Health, USA). Photos in the left are representative of 4 independent experiments and were obtained in a phase contrast microscope (EvosM500, ThermoFisher, Waltham, MA, USA), in 10× objective. Bars corresponds to 10 µm. In the right is the graph with data obtained after four different experiments. Results are expressed as media ± SD (*n* = 4). Statistical significance was calculated in GraphPad Prism 8.0 (San Diego, CA, USA) using analyses of variance (ANOVA) followed by Tukey post-test with *p* < 0.01 (*) when comparing LPS-activated cells treated with LASSBios and LPS-activated cells treated with vehicle.

As we observed a reduction in the number of leukocytes that migrated to SAP, we decided to assess whether this effect could be due to a direct action of LASSBios on cells, thus affecting their migratory capacity. Therefore, LASSBios were incubated with J774.A1 cells and their capacity in affect the wound healing of cells were evaluated after 24 h incubation. Figure 11 (left images) is a representative group of photos obtained at 0 or 24 h after LPS activation and showed that J774.A1 cells migrated into the wound area of the well. The values obtained in these groups were considered as 100% closed area. As can be seen in Figure 11 (right graph), only LASSBio-2039 significantly affected the migratory capacity of cells, thus reducing the percentage of area that became closed after 24 h incubation. 

## 3. Discussion

In this study, we aimed to evaluate the anti-inflammatory effects of three new molecules (named LASSBio-2039, LASSBio-2040 and LASSBio-2041). The advantage of these structures is based on the presence of a *N*-acylhydrazone (NAH) subunit, considered a privileged subunit [10] used for discovering new drug candidates [11].

Formalin injection into the hind paw causes a biphasic response, with the first phase (or neurogenic phase) occurring by an activation of nociceptors present in unmyelinated axons and the second phase (or inflammatory phase) occurring due to the release of mediators (i.e., histamine, prostaglandin, and serotonin) that can sensitize sensory neurons [22]. Drug-mediated reduction in paw licking occurs differently in both phases. Non-steroidal anti-inflammatory drugs act preferentially in the second phase through a peripheral action, whereas opioids act through a central action, inhibiting both phases [23,24,25]. Therefore, this model becomes suitable for the assessment of non-inflammatory or inflammatory pain. Our results showed that pre-treatment with LASSBio-2039 reduced the licking time in both phases and the result in the second phase suggests possible anti-inflammatory activity. 

The remarkable effects of LASSBio-2039, even when compared with the original compound, LASSBio-1760, propelled us to continue investigating the anti-inflammatory effects. In this regard, we used the carrageenan-induced inflammation into the subcutaneous air pouch (SAP). This model is suitable for evaluating the local inflammatory response in vivo. Carrageenan injected into the SAP stimulates an inflammatory response with influx of leukocytes, formation of exudate and accumulation of inflammatory mediators [10]. The migration of cells to the inflammatory site is involved in the development of several diseases. Therefore, inhibiting leukocyte recruitment enables effective control of the inflammatory process [26]. Pre-treatment with LASSBios caused a significant reduction in leukocyte migration. Some possibilities may be involved with this effect. Among them, the inhibition in the production or release of mediators involved with the chemotaxis process or even an action of LASSBios directly on the cells, preventing them from migrating to the inflammatory site.

The production of inflammatory mediators also stimulates the increase in vascular permeability. This step causes the passage of fluids and plasma proteins from the bloodstream to the tissue [27]. Our data showed that all three substances reduced the protein present in the exudate. This effect suggests that LASSBios may be acting by inhibiting the increase in vascular permeability, preventing the proteins from passing into the tissue. According to Lampugnani and collaborators [28], mediators induce the formation of radial stress fibers and the contraction of actomyosin, and this can result in the retraction of the intercellular junction, allowing the passage of fluids and plasma proteins from plasma to tissues. So, our results could suggest a direct effect against cells located in vascular lineage prevent them to contract, thus reducing protein leakage to tissue. 

Interleukin-1β (IL-1β) participates in the inflammatory response through the activation of several molecules such as the cyclooxygenase-2 (COX-2) enzyme, nitric oxide (NO) and endothelial adhesion molecules that will act in the maintenance of inflammatory response [29,30]. Pre-treatment mice with LASSBios reduced the production of the cytokine in the exudate. The inhibition could lead to a decrease in the migration of cells to the inflammatory site and, therefore, a decrease in the arrival of activated cells, producing other mediators and recruiting more cells. This hypothesis is in accordance with Theofilis and collaborators [31] who found that cytokines can influence the expression of adhesion molecules that participate in the process of leukocyte diapedesis. Thus, controlling the production of this cytokine brings benefits for the treatment of various inflammatory diseases [32].

The production of NO plays a fundamental role in the development and maintenance of the inflammatory process. The increase in iNOS (inducible nitric oxide synthase enzyme) expression also occurs in response to activation by lipopolysaccharide (LPS) and the NO produced is one of the key molecules in the pathogenesis of several diseases, so the inhibition of this mediator becomes the target of several anti-inflammatory proposals [33]. Our results show that LASSBios significantly reduced the concentration of NO in the inflammatory exudate. It could be that once there was a reduction in the number of leukocytes, there would also be a reduction in the production of NO. However, the percentage of reduction in NO concentration is not proportional to the reduction in the number of cells that migrated. In this sense our hypothesis is that it may be that the inhibition observed in NO production could be due to the direct effect of LASSBios on the cells, either by inhibiting the production or the activity of the iNOS.

Among the substances that activate macrophages is LPS, which activates Toll-like receptor triggering production of a series of mediators [34,35]. Our data show that all LASSBios reduced IL-1β production by LPS-activated cells. Our next step aimed to analyze the effects of LASSBios on NO production in LPS-activated macrophages. Our data indicate that only LASSBio-2039 reduced the NO production by activated macrophages. After 8 h of activation with LPS, there is a peak in the expression of the iNOS enzyme and that, despite this, the production of NO and this production remains for 24 h [36]. So, we evaluated the effect of LASSBios on NO production after 8 h of LPS activation; however, none of the LASSBios affected NO production. These results together suggest that the action of inhibiting the production of NO caused by LASSBio-2039 can happen through the reduction in the expression of iNOS and not by a reduction in the activity of this enzyme.

After obtaining positive results in reducing the production of inflammatory mediators in vitro, we choose to evaluate whether incubation with LASSBios would influence the process of cell migration. Our assay is advantageous because it mimics the process of cell migration in vivo, in addition to being considered a simple and inexpensive technique to analyze this process [37]. Only incubation with LASSBio-2039 inhibited the macrophage migration process in vitro. This result could be explained by the role of LASSBios in the inhibition of NO that is involved with the SRC-FAK pathway. Macrophages activated with LPS initiate the synthesis of a series of mediators, including the iNOS enzyme responsible for the synthesis of NO. The NO produced actively participates in the SRC-FAK cascade (steroid receptor co-activator-focal adhesion kinase). Studies indicate that this cascade is linked to the process of macrophage mobility, influencing their migratory capacity [38,39]. By understanding the importance of the SRC-FAK cascade in the migration process and how this cascade is highly dependent on NO, our hypothesis is that the effect caused by LASSBio-2039 to reduce cell migration may be occurring due to the reduction caused in the production of NO.

In 2011, LASSBio-1524 was synthesized with the aim of being an inhibitor of the IKK-β enzyme and this was confirmed from structure-based drug design trials. IKK-β is an important enzyme that participates in the activation of the signaling pathway of the nuclear transcription factor kappa B (Nf-κB), the gene responsible for the transcription of several mediators and enzymes that participate in the development and maintenance of the inflammatory process [7]. In 2016, it was observed not only LASSBio-1524, but also LASSbio-1760 pronounced anti-inflammatory activity through reduced cell migration, reducing NO and TNF-α production. A reduction in the expression of phosphorylated Nf-κB suggested that the anti-inflammatory effects of these compounds occur through the inhibition of the Nf-κB signaling pathway [8]. The structural modifications performed for the synthesis of LASSBio-2039, LASSBio-2040 and LASSBio-2041 did not influence the anti-inflammatory activity presented by these compounds. When comparing all three new LASSBios with LASSBio-1524 or LASSBio-1760, only the LASSBio-2039 presented a significant effect when compared with the original molecule (LASSBio-1760). As the molecular target of LASSBio-1524 and LASSBio-1760 is the enzyme that participates in the activation of the Nf-κB pathway, we can assume that the LASSBios tested in this study also act on the same molecular target. This signaling pathway can be activated by the cytokine IL-1β and, once activated, initiates the synthesis of iNOS responsible for producing NO. We observed a reduction in the production of the cytokine, which may have decreased the activation of the Nf-κB signaling pathway, which may cause a reduction in leukocyte migration, in NO, in vascular permeability and a consequent reduction in the concentration of protein in the inflammatory exudate.

The modifications carried out in all three new substances evaluated in this study demonstrated an absence of toxic effects observed in nitroaromatic derivatives [12]. These changes lead to changes in the position of the nitro group to a meta-and ortho-position (in LASSBios-2040 and 2041, respectively). It is also known that the presence of β-substituted naphthyl group confers susceptibility to metabolism with the formation of toxic metabolites [14]. So, the exchange of this group by a α-naphthyl group (in LASSBio-2039) may have influenced this *N*-acylhydrazone to act differently from the others, indicating LASSBio-2039 a pronounced activity in relation to LASSBio-2040 and LASSBio-2041. It is worth noting that the modification carried out in LASSBio-2039 in comparison to LASSBio-1760 resulted in a more pronounced effect, indicating that the addition of the α-naphthyl group resulted in an increase in activity.

It is also important to emphasize that unlike what is observed with anti-inflammatory drugs already on the market, the substances evaluated in this study did not demonstrate toxic effects, either for changes in the blood count, bone marrow, organs, or behavior, which makes them promising substances for testing in chronic disease models as yet untreated.

Taken together, the data demonstrated in this study suggest LASSBio-2039 with a significant anti-inflammatory profile in pre-clinical models of acute inflammation could be developed as a new substance that can be used as a source for the synthesis of new prototypes. 

## 4. Materials and Methods

### 4.1. Chemistry

Reagents and solvents were purchased from commercial suppliers and have not been purified. Melting points were determined in a Quimis Q340.23 apparatus and are uncorrected. ^1^H-NMR and ^13^C-NMR spectra were recorded on Bruker AC-200, Bruker DRX-300 and Varian MR-400 (coupling constant (*J*) values were given in Hertz). Infrared spectra (IR) were carried out in the spectrophotometer apparatus Fourier transform IR Nicolet 6700 FT-IR using tablets of potassium bromide (KBr). Purity of the final product was determined by high-performance liquid chromatography (HPLC) on Shimadzu LC-20AD with Kromasil 100–5 C18 column (4.6 mm × 250 mm), and Detector SPD-M20A (diode array). Analyte quantification was performed using a standardized wavelength, 254 nm, and acetonitrile and water 60% were used as the mobile phase.

Synthetic methodologies used to prepare methyl 1-naphthoate have been carefully described in previously published studies [15,16]. Moreover, 2-naphthohydrazide was prepared as previously described by Cordeiro et al. [8,9]. All the spectroscopical data can be accessed in the Supplementary Material.

### 4.2. Synthesis of 1-Naphthohydrazide (8)

Methyl 1-naphthoate (**7**) (1.4 g, 7.5 mmol) was solubilized in 50 mL of absolute ethanol in a round bottom flask. Then, 5 equivalents of hydrazine hydrate 100% (1.88 mL, 37.6 mmol) were slowly added under magnetic stirring. The mixture was refluxed for 6 h, then volume was partially reduced under reduced pressure. To the flask was added crushed ice and 10 mL of cold water, with precipitate formation. 1-naphthohydrazide (8) (11.6 g, 83%) was isolated by vacuum filtration in a Büchner funnel.

^1^H-NMR (300 MHz, DMSO-*d_6_*) δ 9.71 (s, 1H, NH), 8.23–8.21 (m, 1H), 8.02–7.96 (m, 2H), 7.58–7.51 (m, 4H), 4.62 (s, 3H, NH_2_); ^13^C-NMR (75 MHz, DMSO*-d_6_*) δ 168.00, 133.35, 133.12, 130.0, 129.96, 128.18, 126.64, 126.23, 125.42, 125.35, 124.99. IR (KBr, cm**^−^**^1^) 3276 (ʋ N-H) 1645 (ν C=O).

### 4.3. General Procedure for the Synthesis of N-Acylhydrazone Derivatives (4) and (5)

In a round-bottom flask containing equimolar amounts of hydrazide (**8** or **9**, 0.3 g, 1.6 mmol) and the desired aromatic aldehyde (0.24 g, 1.6 mmol), three drops of 20% aq. HCl and 20 mL of absolute ethanol were added. The mixture was stirred at room temperature for three hours; however, in a few minutes intense formation of precipitates was already visible. The volume of ethanol was partially reduced under reduced pressure and then crushed ice and cold water were added to the flask. Finally, the solid was collected by vacuum filtration in a Büchner funnel.

4-((*E*)-(1-naphthoylimino)methyl)phenylboronic acid (LASSBio-2039, 3): White amorphous solid; 92%; m.p. - °C. ^1^H-NMR (300 MHz, DMSO-*d_6_*) δ 12.07 (s, NH), 8.36 (s, 1H), 8.21 (s, 2H, BOH_2_), 8.10 (d, *J* = 7.8 Hz, 1H), 8.04–8.01 (m, 1H), 7.90 (d, *J* = 7.9 Hz, 2H), 7.78–7.72(m, 3H), 7.64–7.59 (m, 4H). ^13^C-NMR (75 MHz, DMSO-*d_6_*) δ 170.73, 164.78, 147.90, 136.38, 135.62, 134.56, 133.17, 132.79, 130.55, 129.96, 128.39, 127.13, 126.49, 126.14, 125.91, 125.02. IR (KBr, cm**^−^**^1^). **Purity** (HPLC): 99.5%.

(*E*)-*N*-(3-nitrobenzylidene)-2-naphthohydrazide (LASSBio-2040, 4): White amorphous solid; 97%; m.p. -°C. ^1^H-NMR (500 MHz, DMSO-*d_6_*) δ 12.32 (s, 1H), 8.61 (s, 1H), 8.57 (s, 2H), 8.27 (d, *J* = 8.0 Hz, 1H), 8.18 (d, *J* = 7.6 Hz, 1H), 8.10–8.06 (m, 2H), 8.02–8.00 (m, 2H), 7.76 (t, *J* = 7.9 Hz, 1H), 7.67–7.61 (m, 2H). ^13^C-NMR (126 MHz, DMSO-*d_6_*) δ 163.49, 148.28, 145.38, 136.26, 134.49, 133.49, 132.09, 130.54, 130.44, 129.02, 128.09, 127.78, 127.04, 124.36, 120.97. IR (KBr, cm^−1^). **Purity** (HPLC): 99.2%. 

(*E*)-*N*-(2-nitrobenzylidene)-2-naphthohydrazide (LASSBio-2041, 5): White amorphous solid; 94%; m.p. -°C. ^1^H-NMR (300 MHz, DMSO-*d_6_*) δ 12.41 (s, 1H), 8.93 (s, 1H), 8.59 (s, 1H), 8.18 (d, *J* = 7.2 Hz, 1H), 8.11–8.00 (m, 5H), 7.84 (t, *J* = 7.7 Hz, 1H), 7.71–7.63 (m, 3H). ^13^C-NMR (75 MHz, DMSO-*d_6_*) δ 163.43, 148.3, 142.99, 134.52, 133.82, 132.09, 130.75, 130.29, 129.02, 128.81, 128.4, 128.24, 128.1, 128.02, 127.77, 127.02, 124.75, 124.38. IR (KBr, cm^−1^). **Purity** (HPLC): 98.8%.

### 4.4. Animals

Swiss Webster mice (25–30 g) were kindly donated by Instituto Vital Brazil (Niterói, Rio de Janeiro, Brazil). Mice were maintained in a room in a light-dark cycle of 12 h, 22 ± 2 °C from 60% to 80% humidity and with food and water provided ad libitum. Animals were acclimatized to the laboratory conditions for at least 1h before each test and were used only once throughout the experiments. All protocols were conducted in accordance with the Guidelines on Ethical Standards for Investigation of Experimental Pain in Animals [40] and followed the principles and guidelines adopted by the National Council for the Control of Animal Experimentation (CONCEA), approved by the Ethical Committee for Animal Research (# 31/19 and 34/19). All experimental protocols were performed during the light phase. Animal numbers per group were kept at a minimum and at the end of each experiment mice were sacrificed by ketamine/xylazine overdose.

### 4.5. Drugs, Reagents and Treatments

Acetylsalicylic acid (ASA), dexamethasone, L-NMMA (L-N^G^-monomethyl arginine), Ara-C (cytosine arabinoside), MTT (3-(4,5-dimethyl-ltiazol-2-yl)-2,5-diphenyltetrazole) and lipopolysaccharide were purchased from Sigma Aldrich (St. Louis, MO, USA). Ethanol and formalin were purchased from Merck Inc. (São Paulo, Brazil). Cytokines kits were purchased from BD Biosciences (Franklin Lakes, NJ, USA), protein kit (Kit Pierce BCA ™ Protein Assay) was purchased from ThermoFisher Scientific, Inc. (Waltham, MA, USA). Morphine sulfate was kindly provided by Cristália (São Paulo, Brazil). 

LASSBio-1524, LASSBio-1760, LASSBio-2039, LASSBio-2040 and LASSBio-2041 were dissolved in dimethylsulfoxide (DMSO) to prepare 100 µmol/mL stock solutions. For use, solutions were prepared from each stock solution using tween as vehicle. Doses of 0.1 to 10 µmol/kg (final volume of 0.1 mL per animal) were administered by gavage and final tween percentage did not exceed 1%. Acetylsalicylic acid, morphine, dexamethasone, and L-NMMA were used as references drugs. The doses of ASA, morphine, dexamethasone, and L-NMMA were chosen based on previous results obtained by our group when it was calculated the ED_50_ or IC_50_, the dose/concentration caused a 50% reduction in the effect in each procedure. The control group was given vehicle (Tween 80, Isofar, Rio de Janeiro, Brazil). All drugs and LASSBios were diluted just before their use. Data for LASSBio-1524 and LASSBio-1760 are original, independent duplicates of past results and have not been published previously.

### 4.6. Cell Culture

The mouse monocyte macrophage J774.A1 (ATCC # TIB-67) was grown in RPMI medium supplemented with 10% fetal bovine serum (from now on, named as RPMI) and kept in a 5% CO_2_ incubator at 37 °C. An exchange of RPMI was carried out until cells reached 90% confluence and exponential growth. On the day of assays, cells were collected by scraping bottles and adhered in 96- or 12-well culture plates (2 × 10^6^ cells/mL). 

### 4.7. In Vitro Toxicity Test (Cell Viability)

In 96-well plates, J774.A1 cells (10^5^/well, final volume of 200 µL) were adhered at 37 °C, 5% CO_2_. After 30 min incubation with LASSBio-1524 or LASSBio-1760 (30 µM), LASSBio-2039, LASSBio-2040 or LASSBio-2041 (0.1, 1 or 10 µM), LPS (1 µg/mL) was added to some groups. After 24 h of incubation (at 37 °C, 5% CO_2_), supernatant was changed and MTT solution (5 mg/mL, 100 µL/well) was added. After 4 h of incubation (at 37 °C, 5% CO_2_), supernatants were discarded and DMSO (100 µL/well) was added to solubilize the MTT-formazan crystals formed [41]. Absorbance was measured at a wavelength of 570 nm. Control groups were composed by cells which received only RPMI plus DMSO.

### 4.8. In vivo Toxicity Test

Different groups of animals received an oral administration of 100 µmol/kg of LASSBios. After 24 h, mice were euthanized with ketamine (50 mg/kg)/xylazine (20 mg/kg). Sample of blood was collected. The femur was removed, the ends were cut, and the bone marrow was washed with 1 mL of saline (NaCl 0.9%) and collected. Both samples of blood and bone marrow were submitted to a complete blood hemogram and cell count, respectively, in an automatic cell counter (PocH-100iV Diff, Sysmex, Kobe, Japan). Signs of acute toxicity, such as behavioral parameters (i.e., convulsion, hyperactivity, sedation, grooming, loss of righting reflexes, or increased or decreased respiration), as well as food and water intake, were observed over a 5-day period after a single oral dose of each substance (100 µmol/kg) administered to a group of ten animals of both sexes. After this period, the animals were sacrificed by ketamine/xylazine overdose, and their stomachs were removed. An incision was made along the great curvature, and the presence of ulcers or perforations and degree of hyperemia was observed and counted.

### 4.9. Formalin-Induced Paw Licking Model

The method was similar to previously described by Hunskaar and Hole [23,24] with modifications [42]. Briefly, mice received an intraplantar injection of formalin (20 µL, 2.5%) in one hind paw. Immediately they were individually placed in a box and the sum of the times each one remained licking the formalin-injected paw was recorded with the aid of a stopwatch at intervals of 5 min (first phase) or 15–30 min (second phase). Mice were pretreated with vehicle, ASA (1100 µmol/kg), morphine (15 µmol/kg), LASSBio-1524 or LASSBio-1760 (30 µmol/kg), LASSBios (1, 10 or 30 µmol/kg) for 1 h before formalin injection.

### 4.10. Carrageenan-Induced Inflammation into the Subcutaneous Air Pouch (SAP)

The protocol was based in Raymundo et al. [43]. A subcutaneous air pouch was induced in the mice’s back through an injection of 10 mL of sterile air. After 3 days, a new injection of 7 mL of sterile air was performed on the animals’ backs. On the 6th day, the animals were orally treated with vehicle, LASSBios (1, 10 or 30 µmol/kg), LASSBio-1524 or LASSBio-1760 (30 µmol/kg) or dexamethasone (6.5 µmol/kg) and after 60 min mice received an injection of saline or carrageenan (0.5%, 1 mL) into the SAP. After 24 h, the animals were euthanized, and the SAP washed with 1 mL of saline. The exudate was collected for leukocyte count and centrifuged at 1500 r.p.m., for 10 min, 4 °C. The supernatant was collected and stored at −20 °C for several dosages (see below).

### 4.11. Quantification of Proteins and Cytokines

To perform the quantification of proteins in the exudate obtained in the BAS the BCA Protein Assay Kit (Thermo Fisher Scientific, Inc., Waltham, MA, USA) was used. Quantification of cytokines was performed in the exudate collected from BAS and in the supernatant of J774.A1 cells, using an immunoenzymatic assay method (ELISA) with specific ELISA kits (BD OptEIA^TM^ Set mouse, B&D, Albuquerque, NM, USA). Protocols were carried out according to the manufacturer’s instructions. 

### 4.12. Quantification of Nitric Oxide (NO) Production

When produced in biological fluids, NO interacts with hemoglobin and decays to nitrate (NO_3_^−^) and when its production occurs in vitro it interacts with oxygen decaying to nitrite (NO_2_^−^). As the technique does not quantify NO_3_^−^, it is necessary to convert the nitrate generated after NO production in vivo to nitrite. The protocol for converting nitrate to nitrite was described by Bartholomew [44] with adaptations made by Raymundo et al. [43]. 

Both the supernatant collected in the NO_3_^−^ to NO_2_^−^ conversion protocol and that collected from cell cultures were mixed, in equal parts, with the Griess reagent [45]. The absorbance was read in a microplate reader (FlexStation, Molecular Devices, San Jose, CA, USA) at 540 nm. The sodium nitrite concentrations were calculated using a standard sodium nitrite curve.

### 4.13. Inducible Nitric Oxide (iNOS) Synthase Activity and NO-Scavenger Activity Assays

J774.A1 cells were plated in 96 well-plated and incubated with vehicle or lipopolysaccharide (LPS, 1 µg/mL). After 8 h incubation, different concentrations (0.1, 1 or 10 µM) of each LASSBio were added to different groups. Twenty-four hours after LPS activation, the supernatants were collected to NO measurement. 

The NO donor, S-nitroso-*N*-acetyl-DL-penicillamine (SNAP, at 1 mM) was incubated with vehicle or LASSBios (10 µM) for 12 h at 37 °C. After incubation, an aliquot of 0.1 mL was used for nitrite measurement as previously cited.

### 4.14. Cell Migration In Vitro

To assess the effect of LASSBios on cell migration in vitro, J774.A1 cells were plated at 1 × 10^6^ cells per well in 12-well plates (in a final volume of 2 mL) and after 3 h a healing was made in the well with the aid of a P20 tip. The wells were washed with RPMI to remove non-adherent cells. In order to inhibit cell proliferation, the anti-mitotic cytosine Arabinoside (AraC; 10^−5^ M, Sigma-Aldrich, USA) was added to wells. The cells were treated with LASSBios (0.1, 1 or 10 µM) and immediately after treatment and after 24 h, photographs of the wells were performed using an EvosM500 microscope (ThermoFisher). The healing area was measured with the aid of the ImageJ software. To obtain the results, three independent experiments were carried out.

### 4.15. Statistical Analysis

The experimental groups of the in vivo models were composed of 6 to 12 animals selected at random. The in vitro experiments were repeated at least three times on different days (and with a different cell lot) and each experimental group was carried out in triplicate. The results were expressed as mean ± standard deviation (S.D.) and through the analysis of variance test (ANOVA), statistical significance was calculated followed by the Bonferroni post-test with the aid of the GraphPad Prisma 8.02 software. *p* values less than 0.05 (* *p* < 0.05) were considered significant.

## 5. Conclusions

Taken together, our data indicate that LASSBio-2039, LASSBio-2040 and LASSB90-2041 present anti-inflammatory effects, demonstrated in an acute model in vivo and in vitro. We can suggest that these substances could be further studied for the development of new drug prototypes.

## Data Availability

All data can be obtained directly with authors.

## Supplementary Materials

The following supporting information can be downloaded at: https://www.mdpi.com/article/10.3390/ijms232113562/s1.

## References

[1] Rotta D., Fassio A., Rossini M., Giollo A., Viapiana O., Orsolini G., Bertoldo E., Gatti D., Adami G. (2020). Osteoporosis in inflammatory arthritides: New perspective on pathogenesis and treatment. Front. Med..

[2] Maspero J., Adir Y., Al-Ahmad M., Celis-Preciado C.A., Colodenco F.D., Giavina-Bianchi P., Lababidi H., Ledanois O., Mahoud B., Perng D.W. (2022). Type 2 inflammation in asthma and other airway diseases. ERJ Open Res..

[3] Qadri M.A.R.M., Khan A., Alshahrani S., Rashid H., Rashid S., Alsaffar R.M., Kamal M.A., Rehman M.U. (2021). Inflammation and alzheimer’s disease: Mechanisms and therapeutic implications by natural products. Mediat. Inflamm..

[4] Sorriento D., Laccarino G. (2019). Inflammation and cardiovascular diseases: The most recent findings. Int. J. Mol. Sci..

[5] Greten F.R., Grivennikov S.I. (2019). Inflammation and cancer: Triggers, mechanisms, and consequences. Immunity.

[6] Kumar D.P., Koka S., Li C., Rajagopal S. (2019). Inflammatory mediators in obesity. Mediat. Inflamm..

[7] Avila C.M., Lopes A.B., Gonçalves A.S., Da Silva L.L., Romeiro N.C., Miranda A.L.P., Sant’Anna C.M.R., Barreiro E.J., Fraga C.A.M. (2011). Structure-based design and biological profile of (*E*)-*N*-(4-Nitrobenzylidene)-2-naphthohydrazide, a novel small molecule inhibitor of IκB kinase-β. Eur. J. Med. Chem..

[8] Cordeiro N.M., Freitas R.C.N., Fraga C.A.M., Fernandes P.D. (2016). Discovery of novel orally active tetrahydronaphthyl-n-acylhydrazones with in vivo anti-TNF-α effect and remarkable anti-inflammatory properties. PLoS ONE.

[9] Cordeiro N.M., Freitas R.C.N., Fraga C.A.M., Fernandes P.D. (2020). Therapeutic effects of anti-inflammatory *N*-acylhydrazones in the resolution of experimental colitis. J. Pharmacol. Exp. Ther..

[10] Duarte D.B., Vasko M.R., Fehrenbacher J.C. (2016). Models of inflammation: Carrageenan air pouch. Curr. Protoc. Pharmacol..

[11] Thota S., Rodrigues D.A., Pinheiro P.S.M., Lima L.M., Fraga C.A.M., Barreiro E.J. (2018). *N*-acylhydrazones as drugs. Bioorg. Med. Chem. Lett..

[12] Hakimelahi G.H., Khodarahmi G.A. (2005). The Identification of toxicophores for the prediction of mutagenicity, hepatotoxicity and cardiotoxicity. J. Iran. Chem. Soc..

[13] Wang Y., Gray J.P., Heck D.E., Laskin D.L., Laskin J.D. (2008). Role of cytochrome P450 reductase in nitrofurantoin-induced redox cycling and cytotoxicity. Free Radic. Biol. Med..

[14] Black J.W., Duncan W.A.M., Shanks R.G. (1997). Comparison of some properties of pronethalol and propranolol. Br. J. Pharmacol..

[15] Houck H.A., Blasco E., Du Prez F.E., Barner-Kowollik C. (2019). Light-stabilized dynamic materials. J. Am. Chem. Soc..

[16] Shindo K., Osawa A., Kasai Y., Iba N., Saotome A., Misawa N. (2007). Hydroxylations of substituted naphthalenes by *Escherichia coli* expressing aromatic dihydroxylating dioxygenase genes from polycyclic aromatic hydrocarbon-utilizing marine bacteria. J. Mol. Catal. B Enzym..

[17] Lima P.C., Lima L.M., Silva K.C.M., Léda P.H.O., Miranda A.L.P., Fraga C.A.M., Barreiro E.J. (2000). Synthesis and analgesic activity of novel *N*-acylarylhydrazones and isosters, derived from natural safrole. Eur. J. Med. Chem..

[18] Rai G., Kenyon V., Jadhav A., Schultz L., Armstrong M., Jameson J.B., Hoobler E., Leister W., Simeonov A., Holman T.R. (2010). Discovery of potent and selective inhibitors of human reticulocyte 15-lipoxygenase-1. J. Med. Chem..

[19] Lacerda R.B., Silva L.L., de Lima C.K.F., Miguez E., Miranda A.L.P., Laufer S.A., Barreiro E.J., Fraga C.A.M. (2012). Discovery of novel orally active anti-inflammatory *N*-phenylpyrazolyl-*N*-glycinyl-hydrazone derivatives that inhibit TNF-α production. PLoS ONE.

[20] Lopes A.B., Miguez E., Kümmerle A.E., Rumjanek V.M., Fraga C.A.M., Barreiro E.J. (2013). Characterization of amide bond conformers for a novel heterocyclic template of *N*-acylhydrazone derivatives. Molecules.

[21] Palla G., Pelizzi C., Predieri G. (1982). Conformational study on *N*-acylhydrazones of aromatic aldehydes by NMR spectroscopy. Gazz. Chim. Ital..

[22] Parada C.A., Tambeli C.H., Cunha F.Q., Ferreira S.H. (2001). The major role of peripheral release of histamine and 5-hydroxytryptamine in formalin-induced nociception. Neuroscience.

[23] Hunskaar S., Fasmer O.B., Hole K. (1985). Formalin test in mice, a useful technique for evaluating mild analgesia. J. Neurosci. Methods.

[24] Hunskaar S., Hole K. (1986). The formalin test in mice: Dissociation between inflammatory and non-inflammatory pain. Pain.

[25] Tassorelli C., Greco R., Wang D., Sandrini G., Nappi G. (2006). Prostaglandins, glutamate and nitric oxide synthase mediate nitroglycerin-induced hyperalgesia in the formalin test. Eur. J. Pharmacol..

[26] Gupta S., Parent C.A., Bear J.E. (2021). The principles of directed cell migration. Nat. Rev. Mol. Cell Biol..

[27] Miskolci V., Klemm L.C., Huttenlocher A. (2021). Cell migration guided by cell-cell contacts in innate immunity. Trends Cell Biol..

[28] Lampugnani M.G., Dejana E., Giampietro C. (2017). Vascular endothelial (VE)-cadherin, endothelial adherens junctions, and vascular disease. Cold Spring Harb. Perspect. Biol..

[29] Mantovani A., Dinarello C.A., Molgora M., Garlanda C. (2019). Interleukin-1 and related cytokines in the regulation of inflammation and immunity. Immunity.

[30] Szalecki M., Malinowska A., Prokop-Piotrkowska M., Janas R. (2018). Interactions between the growth hormone and cytokines—A review. Adv. Med. Sci..

[31] Theofilis P., Sagris M., Oikonomou E., Antonopoulos A.S., Siasos G., Tsioufis C., Tousoulis T. (2021). Inflammatory mechanisms contributing to endothelial dysfunction. Biomedicines.

[32] Madej M.P., Töpfer E., Boraschi D., Italiani P. (2017). Different regulation of interleukin-1 production and activity in monocytes and macrophages: Innate memory as an endogenous mechanism of IL-1 inhibition. Front. Pharmacol..

[33] Cinelli M.A., Do H.T., Miley G.P., Silverman R.B. (2020). Inducible nitric oxide synthase: Regulation, structure, and inhibition. Med. Res. Rev..

[34] Olona A., Hateley C., Muralidharan S., Wenk M.R., Torta F., Behmoaras J. (2021). Sphingolipid metabolism during Toll-like receptor 4 (TLR4)-mediated macrophage activation. Br. J. Pharmacol..

[35] Ciesielska A., Matyjek M., Kwiatkowska K. (2021). TLR4 and CD14 trafficking and its influence on LPS-induced pro-inflammatory signaling. Cell. Mol. Life Sci..

[36] Pautz A., Li H., Kleinert H. (2021). Regulation of NOS expression in vascular diseases. Front. Biosci. Landmark Ed..

[37] Liang C.C., Park A.Y., Guan J.L. (2007). In Vitro scratch assay: A convenient and inexpensive method for analysis of cell migration in vitro. Nat. Protoc..

[38] Gage M.C., Thippeswamy T. (2021). Inhibitors of Src family kinases, inducible nitric oxide synthase, and NADPH oxidase as potential CNS drug targets for neurological diseases. CNS Drugs.

[39] Cui S., Wu Q., Wang J., Li M., Qian J., Li S. (2019). Quercetin inhibits LPS-induced macrophage migration by suppressing the iNOS/FAK/paxillin pathway and modulating the cytoskeleton. Cell Adhes. Migr..

[40] Zimmermann M. (1983). Ethical guidelines for investigation of experimental pain in conscious animals. Pain.

[41] Mosmann T. (1983). Rapid colorimetric assay for cellular growth and survival: Application to proliferation and cytotoxicity assays. J. Immunol. Methods.

[42] Gomes N.M., Rezende C.M., Fontes S.P., Matheus M.E., Fernandes P.D. (2007). Antinociceptive activity of Amazonian Copaiba oils. J. Ethnopharmacol..

[43] Raymundo L.J.R.P., Guilhon C.C., Alviano D.S., Matheus M.E., Antoniolli A.R., Cavalcanti S.C.H., Alves P.B., Alviano C.S., Fernandes P.D. (2011). Characterisation of the anti-inflammatory and antinociceptive activities of the *Hyptis pectinata* (L.) Poit essential oil. J. Ethnopharmacol..

[44] Bartholomew B. (1984). A rapid method for the assay of nitrate in urine using the nitrate reductase enzyme of *Escherichia coli*. Food Chem. Toxicol..

[45] Green L.C., Wagner D.A., Glogowski J., Skipper P.L., Wisnok J.S., Tannenbaum S.R. (1982). Analysis of nitrate, nitrite, and [^15^N]nitrate in biological fluids. Anal. Biochem..

