# Supplementary figures and images for: Novel Regioisomeric Analogues of Naphthyl-N-Acylhydrazone Derivatives and Their Anti-Inflammatory Effects
Source: Int J Mol Sci. 2022 Nov 5;23(21):13562. doi: 10.3390/ijms232113562 (PMC9657883; doi:10.3390/ijms232113562)

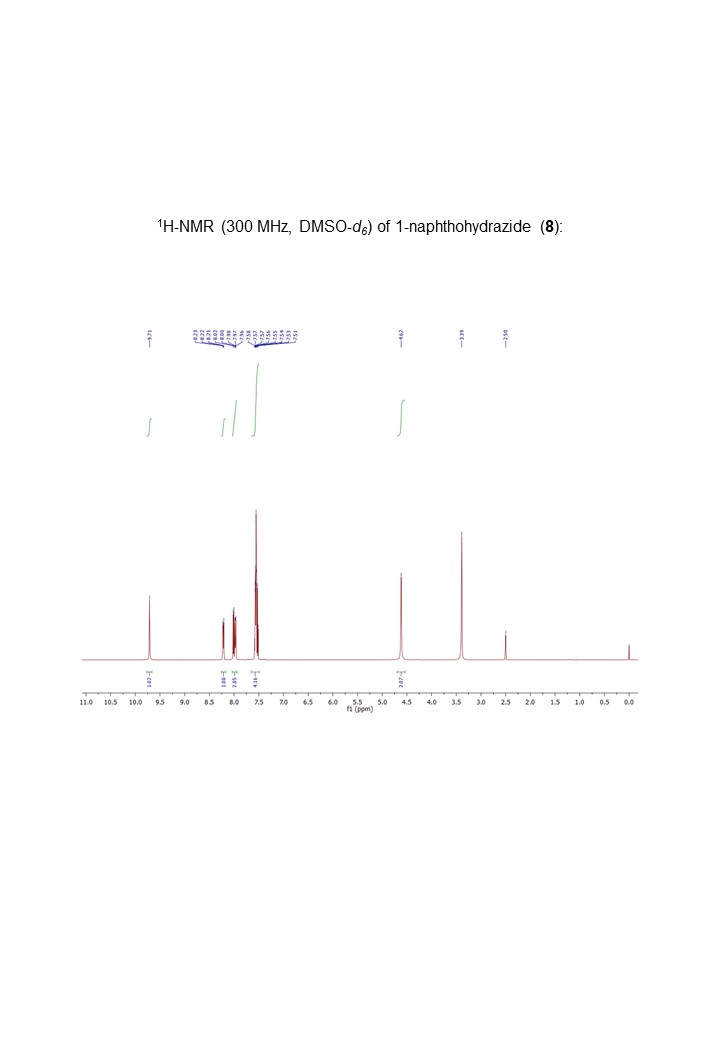

Supplement: Supplementary file 1 [file ijms-23-13562-s001.zip › Slide1.JPG]

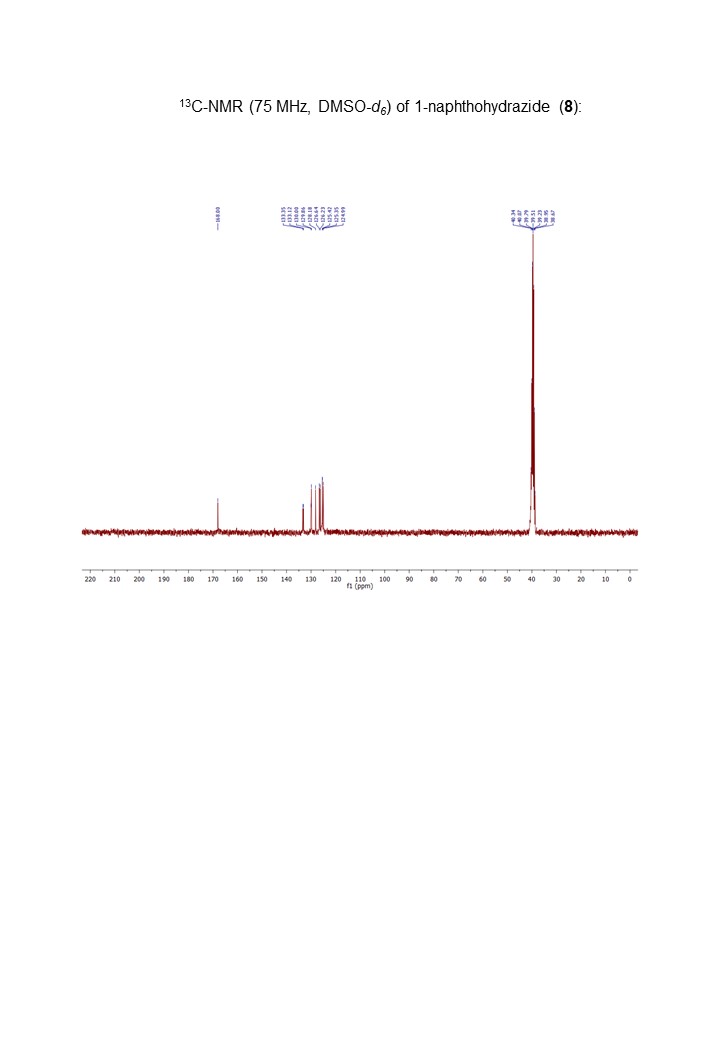

Supplement: Supplementary file 1 [file ijms-23-13562-s001.zip › Slide2.JPG]

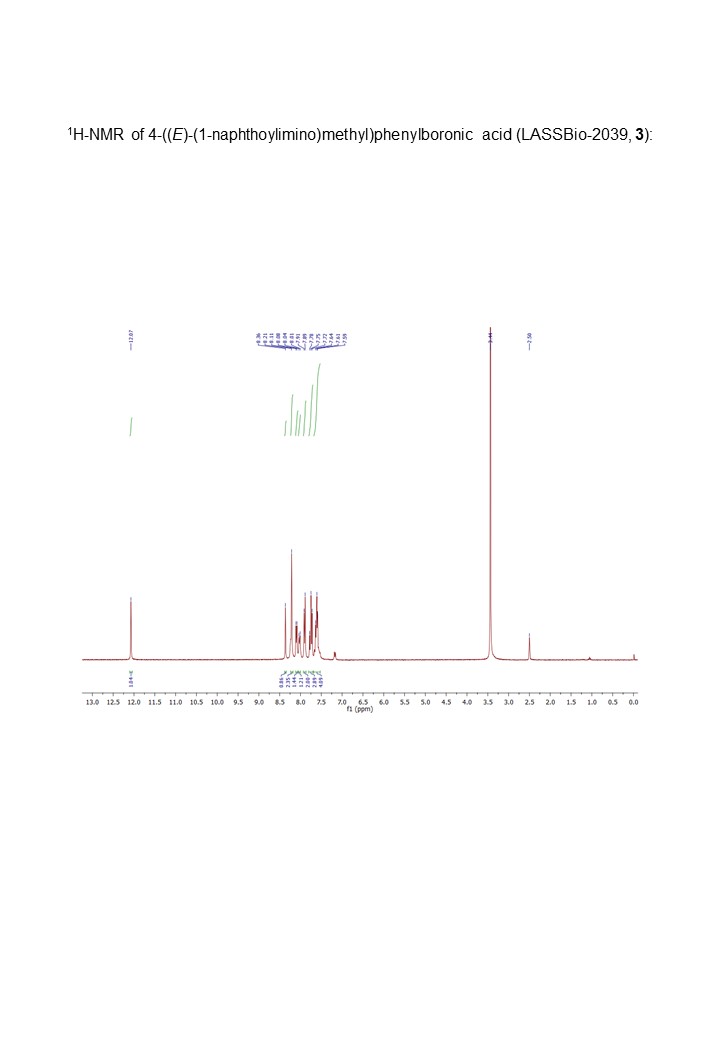

Supplement: Supplementary file 1 [file ijms-23-13562-s001.zip › Slide3.JPG]

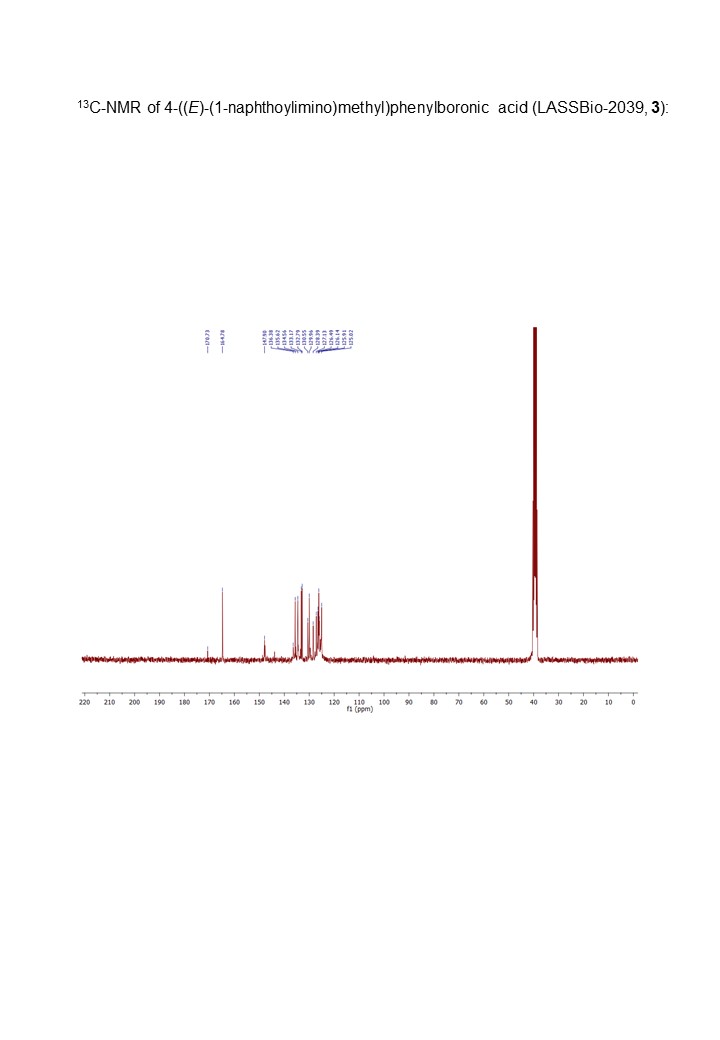

Supplement: Supplementary file 1 [file ijms-23-13562-s001.zip › Slide4.JPG]

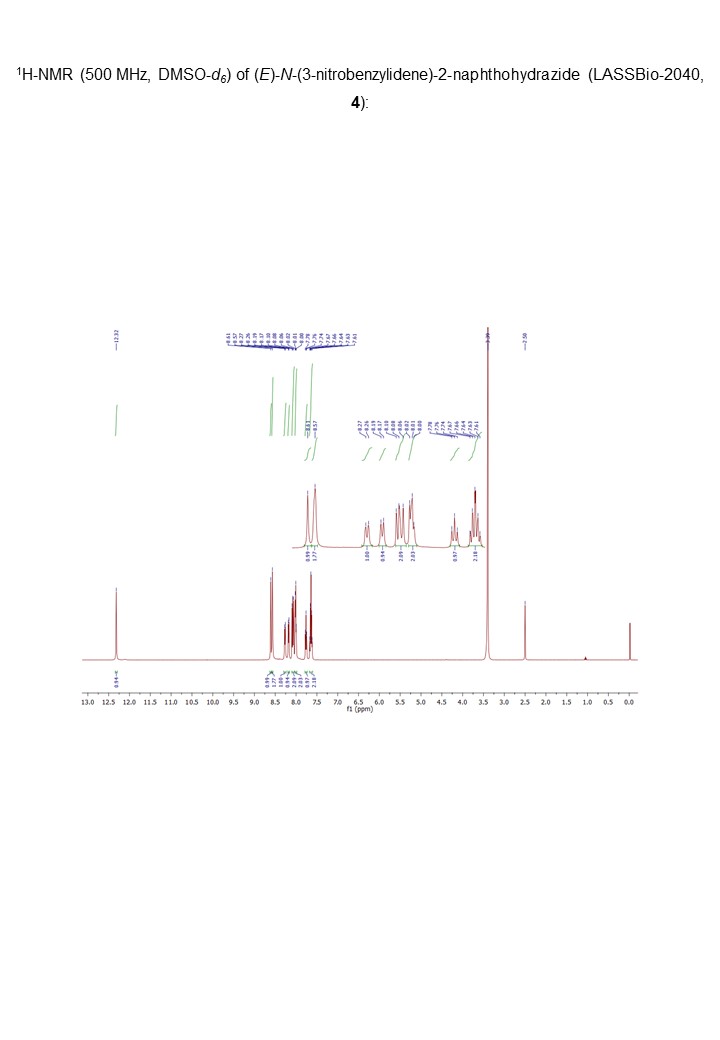

Supplement: Supplementary file 1 [file ijms-23-13562-s001.zip › Slide5.JPG]

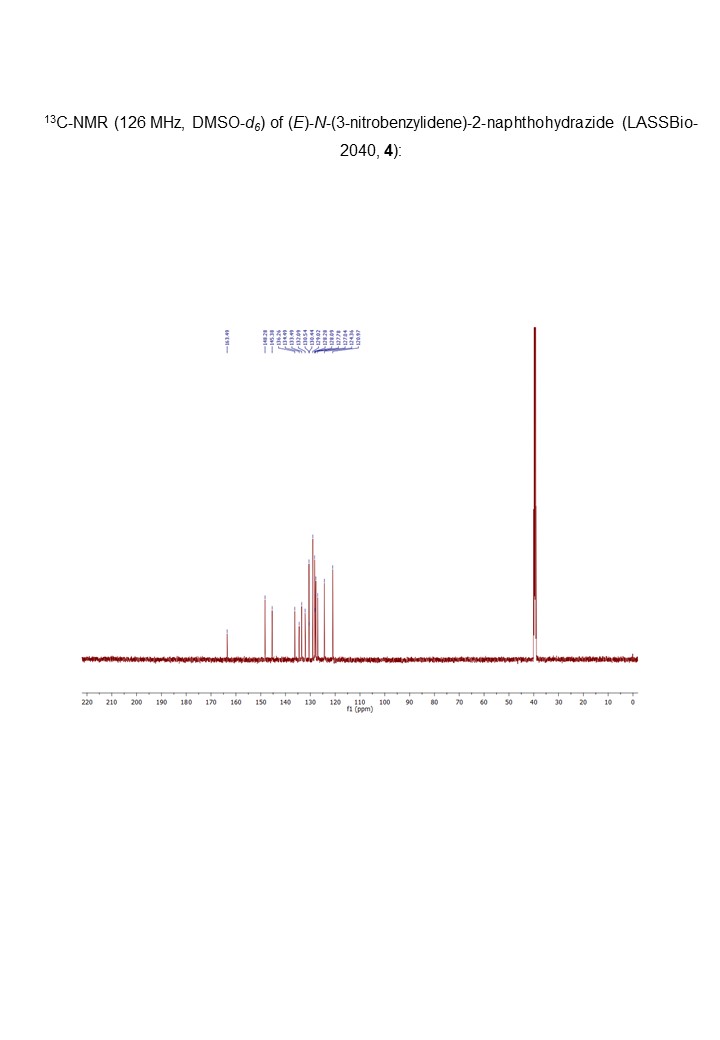

Supplement: Supplementary file 1 [file ijms-23-13562-s001.zip › Slide6.JPG]

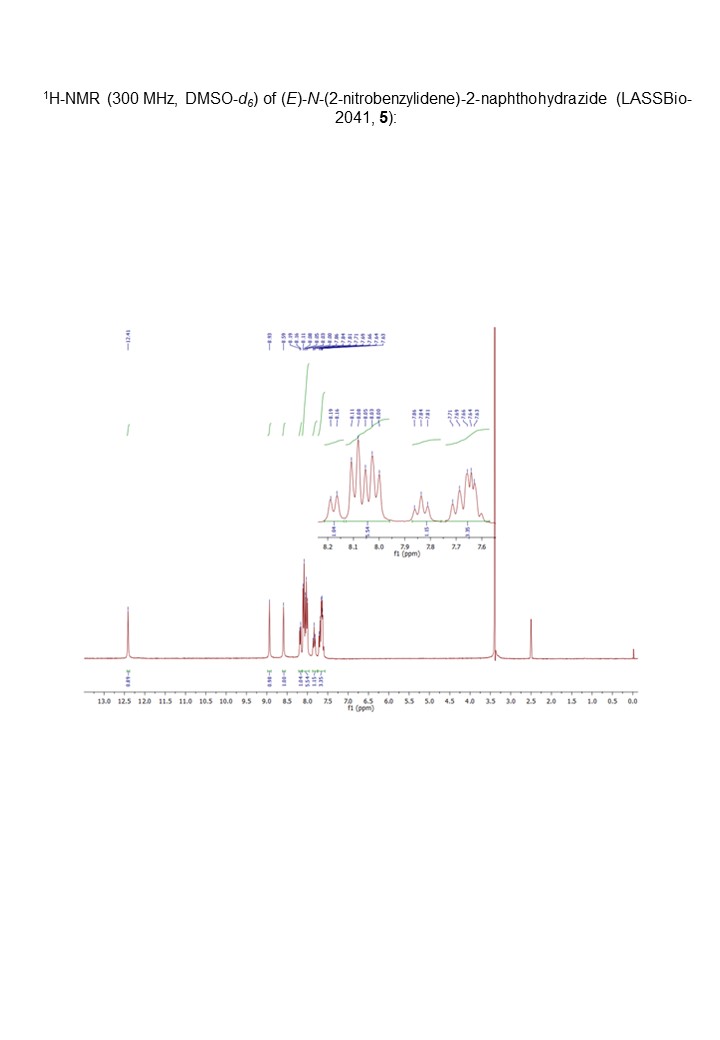

Supplement: Supplementary file 1 [file ijms-23-13562-s001.zip › Slide7.JPG]

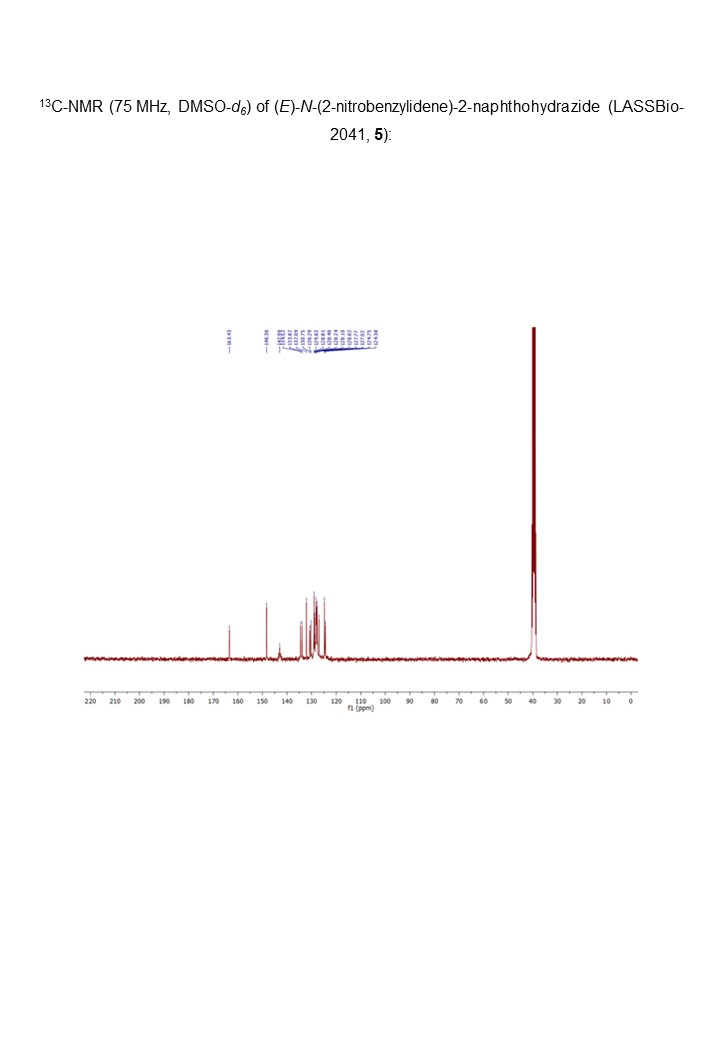

Supplement: Supplementary file 1 [file ijms-23-13562-s001.zip › Slide8.JPG]
